# Stroke risk prediction using machine learning: a prospective cohort study of 0.5 million Chinese adults

**DOI:** 10.1093/jamia/ocab068

**Published:** 2021-05-09

**Authors:** Matthew Chun, Robert Clarke, Benjamin J Cairns, David Clifton, Derrick Bennett, Yiping Chen, Yu Guo, Pei Pei, Jun Lv, Canqing Yu, Ling Yang, Liming Li, Zhengming Chen, Tingting Zhu, the China Kadoorie Biobank Collaborative Group

**Affiliations:** 1 Clinical Trial Service Unit and Epidemiological Studies, Nuffield Department of Population Health, University of Oxford, Oxford, UK; 2 Department of Engineering Science, University of Oxford, Oxford, UK; 3 Medical Research Council, Population Health Research Unit, University of Oxford, Oxford, UK; 4 Oxford-Suzhou Centre for Advanced Research, Suzhou, China; 5 Chinese Academy of Medical Sciences, Beijing, China; 6 Department of Epidemiology and Biostatistics, School of Public Health, Peking University Health Sciences Center, Beijing, China

**Keywords:** stroke, cardiovascular diseases, machine learning, risk assessment, China

## Abstract

**Objective:**

To compare Cox models, machine learning (ML), and ensemble models combining both approaches, for prediction of stroke risk in a prospective study of Chinese adults.

**Materials and Methods:**

We evaluated models for stroke risk at varying intervals of follow-up (<9 years, 0–3 years, 3–6 years, 6–9 years) in 503 842 adults without prior history of stroke recruited from 10 areas in China in 2004–2008. Inputs included sociodemographic factors, diet, medical history, physical activity, and physical measurements. We compared discrimination and calibration of Cox regression, logistic regression, support vector machines, random survival forests, gradient boosted trees (GBT), and multilayer perceptrons, benchmarking performance against the 2017 Framingham Stroke Risk Profile. We then developed an ensemble approach to identify individuals at high risk of stroke (>10% predicted 9-yr stroke risk) by selectively applying either a GBT or Cox model based on individual-level characteristics.

**Results:**

For 9-yr stroke risk prediction, GBT provided the best discrimination (AUROC: 0.833 in men, 0.836 in women) and calibration, with consistent results in each interval of follow-up. The ensemble approach yielded incrementally higher accuracy (men: 76%, women: 80%), specificity (men: 76%, women: 81%), and positive predictive value (men: 26%, women: 24%) compared to any of the single-model approaches.

**Discussion and Conclusion:**

Among several approaches, an ensemble model combining both GBT and Cox models achieved the best performance for identifying individuals at high risk of stroke in a contemporary study of Chinese adults. The results highlight the potential value of expanding the use of ML in clinical practice.

## INTRODUCTION

Stroke is a leading cause of death and disability worldwide, with about three-quarters of all stroke cases occurring in low- and middle-income countries (LMICs).^1^ China has the largest stroke burden in the world, and accounts for approximately one-third of global stroke mortality with 34 million prevalent cases and 2 million deaths in 2017.^2^^,^^3^ Current guidelines for primary prevention of stroke advocate the use of risk prediction models to identify individuals at high risk of cardiovascular disease (CVD) including stroke.^4–6^ It has been estimated that with early intervention, half of all strokes could be prevented by controlling modifiable risk factors in such individuals.^7^

Commonly used risk scores include the Pooled Cohort Equations^8^ and QRISK^9–11^ for CVD, as well as the Framingham Stroke Risk Profile^12^^,^^13^ for stroke. Such risk scores are typically derived using Cox proportional hazards models and have been validated mainly in high-income countries (HICs).^14–16^ However, the clinical utility of such models for risk prediction of stroke in contemporary populations of LMICs such as China is uncertain, and novel risk scores should be developed for use in such populations.^17–19^

Machine learning (ML) techniques have been increasingly used in recent years for a variety of healthcare applications, and have demonstrated superior predictive value compared with traditional Cox models for predicting risk of stroke or overall CVD.^20–23^ However, these ML models have still not been widely adopted in clinical practice and little is known about the utility of such risk scores for prediction of stroke risk in a contemporary Chinese population.^24^

## OBJECTIVES

The aims of this study were to (i) compare Cox and ML models for prediction of risk of stroke in China at varying intervals of follow-up (ie, stroke within 9 years, 0–3 years, 3–6 years, 6–9 years); (ii) identify individuals for whom ML models might be superior to conventional Cox-based approaches for stroke risk prediction; and (iii) develop and evaluate an ensemble model combining both approaches to identify individuals at high risk of stroke.

## MATERIALS AND METHODS

### Study population

The China Kadoorie Biobank (CKB)^25^^,^^26^ is a prospective cohort study of 512 726 participants enrolled from 10 geographically diverse areas (5 urban, 5 rural) of China in 2004 to 2008. In each area, all permanent residents without disability aged 35–74 years were invited to participate. An interviewer-administered electronic questionnaire was used to collect data on sociodemographic factors, lifestyle factors (eg, smoking, alcohol, dietary habits), medical history and current medication, and physical activity. Physical measurements included height, weight, hip and waist circumference, bio-impedance, blood pressure, and heart rate. All participants provided a blood sample, and random blood glucose tests were conducted to screen for diabetes.^26^^,^^27^ All follow-up data were collected by linkage to death registries, established registries of major diseases, and health insurance records (covering >97% of participants); local residential records; and annual home visits for uninsured participants through January 1, 2018.^26^ All stroke cases were verified and adjusted by trained medical staff using the International Classification of Diseases 10th revision (ICD-10) (Supplementary Methods S1).^28^

The present analyses were restricted to 205 293 men and 298 549 women with no prior history of stroke or transient ischemic attack at baseline (8884 individuals excluded), and all incident cases of first stroke that were recorded for up to 9 years after the baseline survey for each individual were included (19 587 strokes in men; 23 647 strokes in women). After data preprocessing, including accounting for missing values, the dataset included 143 risk factor indicators in addition to incident stroke cases and a time-to-event for each stroke event (Supplementary Methods S2). Ethical approval for CKB was obtained from the Oxford University Tropical Research Ethics Committee and the Chinese Center for Disease Control and Prevention Ethical Review Committee, and all participants provided written informed consent.

### Model development and validation

**Figure 1.**
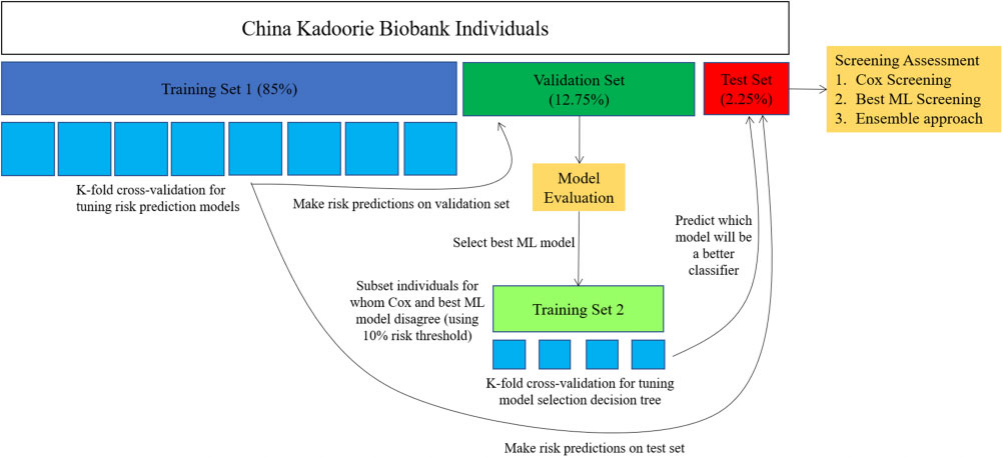
Cox and machine learning (ML) model development and validation. All analyses were performed separately for men and women. Included individuals were divided into a training set (85%), validation set (12.75%), and test set (2.25%). Risk prediction models were developed in the training set and assessed in the validation set, with a best ML model selected. The traditional Cox model and best ML model were then used for screening high-risk individuals in the validation set using a 10% predicted risk threshold. A second training set was created from a subset of the validation set wherein the Cox model and best ML model disagreed on risk classification, and a decision tree was trained to predict which model would yield a better risk classification for each individual. Screening approaches, including a (i) Cox-only approach, (ii) best ML-only approach, and (iii) an ensemble approach, were assessed and compared using the held-out test set.

CKB individuals were randomly assigned to a training set (85%; 174 498 men with 16 649 strokes; 253 766 women with 20 100 strokes), a validation set (12.75%; 26 174 men with 2467 strokes; 38 065 women with 3014 strokes), and test set (2.25%; 4620 men with 471 strokes; 6718 women with 533 strokes), with all subsequent analyses performed separately by sex (Figure 1). Cox, random survival forest (RSF), logistic regression (LR), support vector machine (SVM), gradient boosted tree (GBT), and multilayer perceptron (MLP) models were derived in the training set for risk prediction of stroke within 9 years of the baseline survey. To explore differences in performance and major risk factors for short-term and long-term risk prediction, models were also derived for follow-up intervals of 0–3 years, 3–6 years, and 6–9 years after baseline. Features were selected and hyperparameters tuned in each model using k-fold cross-validation within the training set (Supplementary Methods S3), and the final models were evaluated in the validation set. All models were benchmarked against the 2017 Framingham Stroke Risk Profile (FSRP),^13^ both with and without recalibration and refitting to the CKB cohort. Atrial fibrillation was not recorded in CKB and was excluded from the FSRP model.

Survival analysis approaches (FSRP, Cox, RSF) differ from binary classifiers (LR, SVM GBT, MLP) in their ability to account for censored individuals and yield time-to-event probabilities for stroke. Consequently, stroke-free individuals who died or were lost to follow-up before 9 years (5.4% of all participants) were included in the training set for development of survival models but not in the binary classification models. These right-censored individuals were further excluded from the validation set and test set for all models, since it was unknown if they could have suffered a stroke within the time interval of interest. Furthermore, while a single Cox and RSF model could be used for risk prediction at all time scales, separate binary classification models were derived for each prediction task.

After comparing model discrimination and calibration, the predictions of the Cox model and best-performing ML model were used to identify individuals at high risk of stroke (defined as having >10% predicted risk of stroke in 9 years) in the validation set. Agreement between the Cox model and best-performing ML model was assessed qualitatively using t-Distributed Stochastic Neighbor Embedding (t-SNE)—an unsupervised, nonlinear technique for visualizing high-dimensional data. A second training set was generated by restricting the validation set to the individuals for whom the Cox model and best ML model disagreed (Table 2), and a decision tree was derived from this training set (Supplementary Methods S3) to predict which model would yield a better classification (ie, “high-risk” if the individual had a stroke or “not high-risk” if the individual did not have a stroke) based on individual-level characteristics. Feature importance for the decision tree was analyzed using the Gini importance metric.^29^ Screening approaches to identify individuals at high risk of stroke were then compared in the test set, including a Cox-only approach, best ML-only approach, and an ensemble approach that used the trained decision tree to select a model in cases of disagreement between Cox and the best ML model.

### Statistical analysis

The Cox and ML models were assessed for risk discrimination and calibration performance in the validation set. Risk discrimination refers to the ability to correctly discriminate between individuals with and without stroke,^30^ and was evaluated using the area under the receiver operating characteristic curve (AUROC), with higher AUROCs indicating better risk discrimination. Calibration refers to the similarity between observed and predicted numbers of stroke events for each predicted risk decile,^30^ and was evaluated using chi-squared test statistics (χ2) from the Hosmer-Lemeshow test (for binary classification models) and Nam-D’Agostino test (for survival models), with lower χ2 values indicating better calibration.^31^^,^^32^ The 95% confidence intervals were estimated for AUROC and χ2 values using 1000 bootstrapped samples from the validation set.

Risk screening approaches were evaluated in the test set, using sensitivity, specificity, positive predictive value (PPV), negative predictive value (NPV), and accuracy. Agreement between approaches was assessed using Cohen’s kappa (κ). For the ensemble screening approach, the decision tree to select between Cox and the best ML model was evaluated for accuracy as well as discriminatory performance using AUROC in the test set.

Statistical analyses were performed using Python version 3.7.0 and R version 3.6.1. Cox models were implemented using the lifelines package^33^ version 0.21.1 with LASSO variable selection performed in R using the glmnet package^34^ version 3.0-2. RSF models were implemented using the ranger package^35^ version 0.12.1. LR, SVM, and GBT models were implemented using scikit-learn toolkit^36^ version 0.19.2, and MLP models were implemented using keras^37^ version 2.3.1. tSNE visualizations were implemented using the Rtsne package version 0.15.^38^

## RESULTS

Among the included study participants, the mean (SD) age was 51.9 (10.6) years and 59% were women (Table 1). During 9 years of follow-up, a total of 43 234 individuals had a first stroke (Supplementary Figure S1). The incidence of stroke was higher in men than in women (9.5% vs 7.9%) and varied by more than 5-fold between the 10 study areas. Compared with those who had no stroke, individuals who had a first stroke were older and more likely to have prior history of CHD, diabetes, or hypertension (Table 1). Overall, men and women had similar proportions with prior history of CHD (2.5% vs 3.0%), diabetes (5.3% vs 6.0%), and use of blood pressure-lowering medication (9.9% vs 11.4%), but the prevalence of current smoking was much higher in men than in women (67.7% vs 3.2%).

**Table 1.** Distribution of established risk factors for stroke in men and women by presence or absence of stroke during follow-up

	Men		Women	
Risk factors in 2017 Framingham Stroke Risk Profile^a^	No Stroke (n = 185 706)	Stroke (n = 19 587)	No Stroke (n = 274 902)	Stroke (n = 23 647)
Age, mean, year	51.8	60.7	50.6	59.6
Current smoking, %	68.5	59.9	3.1	4.8
Coronary heart disease, %	2.1	6.4	2.5	9.4
Age 65 yrs+, %	13.8	39.9	10.7	34.0
Diabetes at age <65 yrs, %	3.7	6.5	4.0	8.1
Diabetes at age 65+ yrs %	1.1	4.8	1.3	6.0
BP-lowering treatment, %	8.6	22.3	10.1	26.3
SBP-untreated, mean, mmHg	130	142	126	138
SBP- treated, mean, mmHg	148	153	150	155

*Note:* “No Stroke” column includes individuals who remained stroke-free until being censored, even if lost to follow-up before 9 years.

a Atrial fibrillation is a part of the Framingham Stroke Risk Profile but was not recorded in CKB.

### Comparisons of cox versus ML models to predict risk of stroke

**Figure 2.**
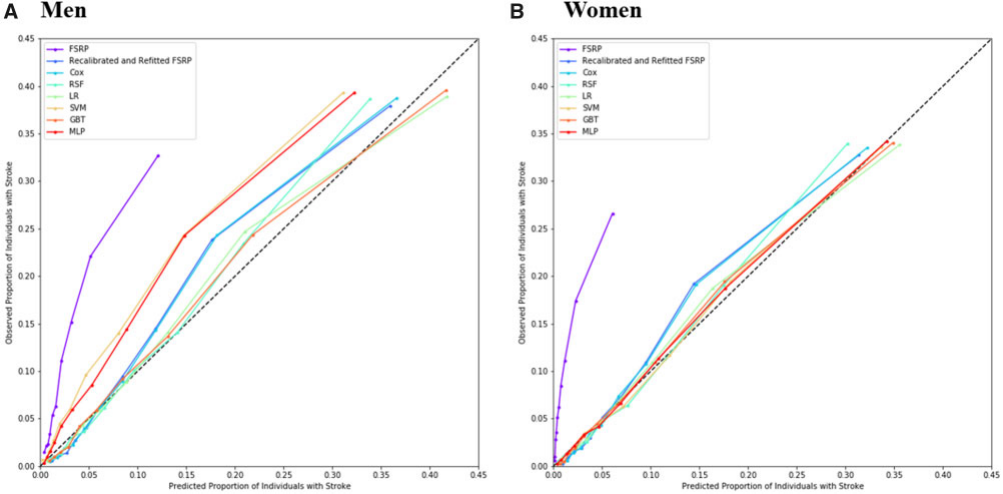
Calibration plots for the 2017 Framingham Stroke Risk Profile (FSRP), a recalibrated and refitted FSRP, Cox, random survival forest (RSF), logistic regression (LR), support vector machine (SVM), gradient boosted tree (GBT), and multilayer perceptron (MLP) models in (A) men and (B) women. Each point represents a decile of predicted risk.

The Cox model and ML models all outperformed the 2017 Framingham Stroke Risk Profile (FSRP) with and without recalibration and refitting and achieved similar discrimination for 9-year risk of stroke, with GBT yielding marginally higher AUROCs than other models for both men and women (Table 2, Supplementary Figure S2). However, calibration performance varied substantially between models. RSF, LR, and GBT all yielded lower χ2 values than Cox models in both men and women, with GBT showing significant improvements in calibration performance (Table 2, Supplementary Figure S3). MLP and SVM (after isotonic regression) were observed to yield good calibration for women but were poorly calibrated for men. Nevertheless, all models were significantly better calibrated than the original FSRP, which severely underestimated stroke risk in the CKB cohort (Figure 2, Supplementary Figure S3). Calibration plots (Figure 2) indicated that calibration for all models was better in women than in men, with models underestimating risk of stroke in men compared with women. Furthermore, deviation from perfect calibration was more extreme in those at highest risk of stroke (Figure 2) and in older individuals (Supplementary Figure S4). Due to its high AUROC and improvements to calibration over the traditional Cox model, GBT was identified as the best-performing ML model and was selected for further risk screening analyses.

**Table 2.** Discrimination and calibration performance for prediction of 9-year risk of stroke. Comparisons included the 2017 Framingham Stroke Risk Profile (FSRP), a recalibrated and refitted FSRP, Cox, random survival forest (RSF), logistic regression (LR), support vector machine (SVM), gradient boosted tree (GBT), and multilayer perceptron (MLP) models

	Men		Women	
Model Type	Discrimination	Calibration	Discrimination	Calibration
	AUROCs	χ^2^	AUROCs	χ^2^
	[95%CI]	[95%CI]	[95%CI]	[95%CI]
FSRP	0.781	5541	0.772	19402
	[0.772-0.790]	[4996-6107]	[0.764-0.780]	[17784-21019]
Recalibrated and refitted	0.824	138	0.825	140
FSRP	[0.816-0.831]	[96-185]	[0.819-0.833]	[97-186]
Cox	0.829	122	0.831	129
	[0.822-0.837]	[83-166]	[0.824-0.838]	[89-172]
RSF	0.826	61	0.832	62
	[0.818-0.834]	[36-90]	[0.824-0.839]	[36-95]
LR	0.831	56	0.832	57
	[0.823-0.838]	[31-86]	[0.825-0.838]	[34-85]
SVM	0.830	712	0.831	24
	[0.823-0.838]	[582-852]	[0.824-0.838]	[11-41]
GBT	0.833	44	0.836	47
	[0.825-0.840]	[24-67]	[0.829-0.843]	[30-69]
MLP	0.831	515	0.833	19
	[0.824-0.839]	[410-627]	[0.826-0.841]	[8-35]

**Figure 3.**
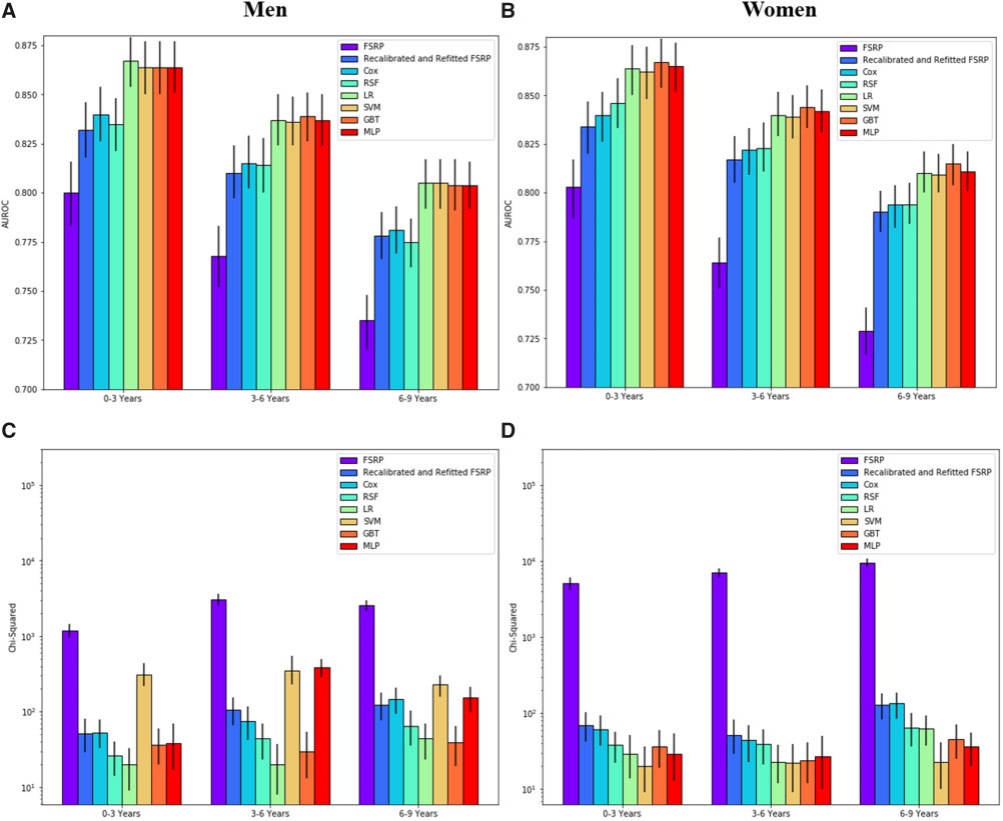
Discrimination (subplots A and B) and calibration (subplots C and D) performance in men and women, respectively, for risk prediction of stroke at various time scales (0–3 years, 3–6 years, 6–9 years after baseline). Comparisons made between the 2017 Framingham Stroke Risk Profile (FSRP), a recalibrated and refitted FSRP, Cox, random survival forest (RSF), logistic regression (LR), support vector machine (SVM), gradient boosted tree (GBT), and multilayer perceptron (MLP) models.

Evaluation of risk prediction models at varying intervals of follow-up (0–3 years, 3–6 years, 6–9 years) demonstrated comparable relative performance between models (Figure 3, Supplementary Tables S1–S3). However, binary classification models (LR, SVM. GBT, and MLP), which were retrained for each prediction task, yielded substantial improvements over survival models (FSRP, Cox, RSF) at all intervals of follow-up and for both sexes (Figures 3A and 3B). The AUROCs for all models decreased monotonically for later intervals of follow-up. For most models, calibration also declined at later intervals of follow-up, but was less sensitive to changes in times scales than discrimination performance (Figures 3C and 3D). At all intervals of follow-up, MLP and SVM (after isotonic regression) had a tendency for poor calibration in men, but were well-calibrated in women. Once again, the FSRP without recalibration and refitting, yielded the worst calibration, substantially underestimating stroke risk at all intervals of follow-up.

GBT remained among the best-performing of the ML models based on discrimination and calibration metrics. Additional analyses of the most important GBT features for later intervals of follow-up (Supplementary Table S4) indicated comparable performance in men and women with emphasis on risk factor indicators related to age, blood pressure, physical activity, and geographic area. However, characteristics, such as retirement and number of children, became relatively more important at later intervals of follow-up (3–6 years and 6–9 years) than at 0–3 years from baseline.

### Prediction of best model for risk screening

After identifying GBT as the best-performing ML model, risk predictions estimated using the Cox and GBT models were used to screen individuals at high risk of stroke in the validation set in order to train a decision tree to select which model to use for a particular individual, given disagreement about the individual’s risk classification. For the purposes of this study, individuals were classified as “high-risk” if they had >10% predicted risk of stroke in 9 years. In cases of disagreement between models, either the Cox or GBT model was identified as the better classifier if it classified an individual with stroke as “high-risk” or classified a stroke-free individual as “not high-risk”.

**Figure 4.**
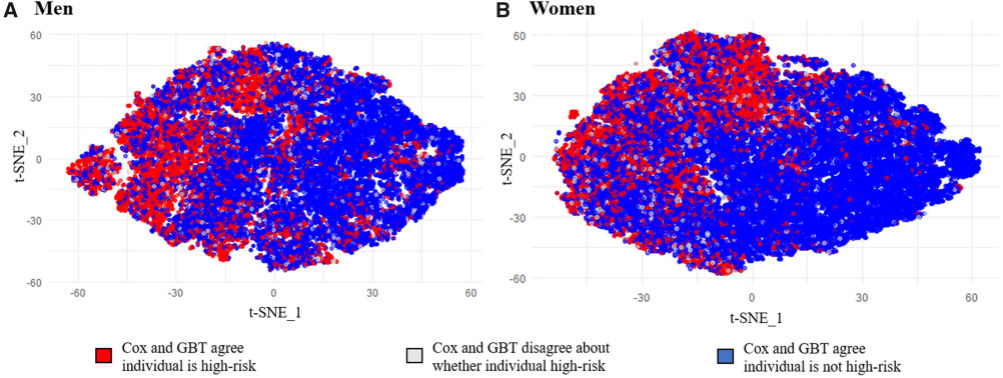
t-Distributed Stochastic Neighbor Embedding (t-SNE) visualizations of CKB individuals in validation set and test set. Individuals are colored by agreement between Cox and GBT risk prediction models for screening of high-risk individuals. High-risk individuals were defined as individuals with >10% predicted 9-yr risk of stroke. t-SNE plots were created using Rtsne package version 0.15 with perplexity = 50, theta = 0.5, and max iterations = 3000.

The t-SNE visualizations of individuals in the validation set and test set (Figure 4) indicated high levels of agreement between both the Cox and GBT models for stroke risk prediction in both men and women. Disagreements occurred in only 5% of men and women, of whom 10% of men and 12% of women would go on to experience a stroke event.

Among the 4281 men and 6441 women in the test set (after excluding stroke-free, right-censored individuals), sex-specific decision trees were used to resolve 590 disagreements (219 in men, 371 in women) and selected GBT as the better classifier 31% of the time (34% for men, 29% for women). Among individuals in the test set for whom the Cox and GBT models disagreed, the decision trees (Supplementary Figures S5–S6) yielded good discrimination for model selection (AUROC: 0.71 in men, 0.74 in women) and correctly selected the better classifier with an accuracy of 73% in men and 70% in women. The most important features for selecting the better classifier included blood pressure, age, and geographic area-related risk factor indicators for both sexes—in addition to additional features reported in the online supplement (Supplementary Table S5).

### Comparison of risk screening approaches

In both men and women, the ensemble approach yielded a higher accuracy (76% in men, 80% in women) and specificity (76% in men, 81% in women) compared with either Cox-only or GBT-only approaches (Table 3). The ensemble approach also yielded the highest PPV in men (26%). The GBT-only approach yielded the highest sensitivity (80% in men, 74% in women), and the Cox-only approach did not provide the best performance using any metric. Differences in performance were incremental for all metrics with overlapping 95% confidence intervals. The confusion matrices for each screening approach are reported for both sexes in the online supplement (Supplementary Table S6). Good agreement was observed between all models with κ values ranging from 0.85 to 0.96 (Supplementary Table S7).

**Table 3.** Summary metrics from screening of high-risk individuals in test set using (i) a Cox-only approach, (ii) a GBT-only approach, and (iii) an ensemble approach in which a decision tree selects between Cox and GBT in cases of disagreement over an individual’s risk classification

	Men			Women		
Metric	Cox-Only	GBT-Only	Ensemble	Cox-Only	GBT-Only	Ensemble
	[95% CI]	[95% CI]	[95% CI]	[95% CI]	[95% CI]	[95% CI]
Sensitivity	76%	80%	76%	68%	74%	67%
	[72%–80%]	[76%–84%]	[72%–80%]	[64%–72%]	[70%–78%]	[64%–71%]
Specificity	75%	74%	76%	80%	78%	81%
	[74%–77%]	[73%–76%]	[75%–78%]	[79%–81%]	[77%–79%]	[80%–82%]
PPV	25%	25%	26%	24%	23%	24%
	[23%–27%]	[23%–28%]	[23%–28%]	[22%–26%]	[21%–25%]	[22%–26%]
NPV	97%	97%	97%	97%	97%	97%
	[96%–97%]	[96%–98%]	[96%–97%]	[96%–97%]	[97%–98%]	[96%–97%]
Accuracy	75%	75%	76%	79%	77%	80%
	[74%–77%]	[74%–76%]	[75%–77%]	[78%–80%]	[76%–78%]	[79%–81%]

## DISCUSSION

In this study, involving almost a 100-fold larger population than the original Framingham Study,^13^ we developed novel risk scores for prediction of stroke in a contemporary Chinese cohort. Previous population-based prospective studies^17–19^ highlighted the need for novel risk scores for use in Chinese adults and proposed new Cox-derived models based on these populations. However, the models derived in the present study were based on a substantially larger (5-fold compared to the China-PAR study^17^) and more contemporary population.

In contrast to previous studies of risk prediction of stroke in Chinese adults, we compared both conventional Cox model-based approaches and ML techniques for risk prediction to assess the potential of ML techniques for improved risk prediction. Consistent with findings for cardiovascular risk prediction,^20^ we demonstrated that ML techniques improved 9-yr risk prediction of stroke over Cox models, with GBT providing the best discrimination and calibration performance. Improvements over the Cox model were particularly evident for binary classification models that predicted stroke at narrower intervals of follow-up (0–3 years, 3–6 years, and 6–9 years from baseline). This may be due to the fact that survival models, such as Cox and RSF are optimized across the overall 9-year follow-up period, while binary classification models are able to be retrained for optimal performance in each particular time interval of interest. All models substantially outperformed the 2017 Framingham Stroke Risk Profile, which greatly underestimated stroke risk in CKB.

While the discrimination improvements of ML over Cox models were marginal, such incremental improvements can translate to meaningful population health benefits. For example, a recent analysis of 100 000 UK adults reported that polygenic risk scores for CVD with improvements of just 0.012 in the C-index could help to prevent 7% more CVD events than conventional risk scores alone.^39^ Moreover, the substantial calibration improvements of ML approaches, such as GBT, over Cox models are highly relevant for clinical practice, in which decisions on initiation of drug treatment may be informed by defined risk thresholds. Contemporary clinical guidelines recommend using absolute risk predictions from Cox models to screen individuals at high risk of stroke, who are then prioritized for initiation of drug treatments. For example, the 2013 guidelines of the American College of Cardiology and the American Heart Association (ACC–AHA)^40^ recommend initiation of statin therapy for those with a ≥ 7.5% 10-year CVD risk as assessed by the Pooled Cohort Equations, while in the UK, the cutoff is ≥10% risk as assessed by QRISK3.^41^ In such settings, underestimation of stroke risk due to poor calibration of models could result in failure to identify high-risk individuals who would benefit from statins or other preventative drug treatments.

Using a threshold of ≥10% 9-year stroke risk, we found that an ensemble approach that combined Cox and GBT models had a higher accuracy, specificity, and PPV for stroke prediction than either the Cox-only or GBT-only approaches. However, such improvements were marginal and warrant assessment for reproducibility in external validation studies. We have provided statistical code in the online Supplementary Material to enable others to replicate these findings in other populations.

In contrast with typical ensemble approaches that use voting or averaging of base model outputs, a major strength of our ensemble approach is its default reliance on the Cox model. Cox model-based approaches to risk prediction are widely used in clinical practice, and their relative simplicity and interpretability have been challenges to the adoption of novel ML-based methods.^42^ We found that the component Cox and GBT models failed to agree on risk prediction for stroke in about 5% of individuals, and of these, our proposed ensemble approach selected GBT as the better-performing model about one-third of the time. This suggests that, in practice, our ensemble approach would override the risk classification of the Cox model for only a small proportion (1%–2%) of individuals. Meanwhile, clinicians could continue to use the output from Cox model-derived scores, without any loss of predictive performance for the vast majority of individuals at high risk of stroke. Rather than changing the existing paradigm of stroke risk prediction, our proposed ensemble approach has been designed as an incremental change to clinical practice, which could help to facilitate more widespread use and trust of ML methods for health risk prediction.^43^

Other barriers to the adoption of complex high-dimensional models for application in clinical practice include the availability of certain risk factor data and the need for regular updating and recalibration of such models. However, as electronic health records (EHRs) become more detailed and widespread, they may mitigate these issues by providing detailed individual-level data and enabling automatic updating and recalibration of complex ML models to local practices.^43^

This study had several limitations. First, atrial fibrillation (AF), which is commonly included in risk scores for CVD and stroke ,^10^^,^^13^ was not recorded in the CKB and could not be included in the models. However, other population-based studies of comparable age groups in China indicated that the prevalence of AF was substantially lower in China than in the Framingham Stroke Risk Profile cohort (1.7% vs 7.1%).^13^^,^^44^ Hence, omission of AF is unlikely to have had a material impact on stroke risk prediction in CKB. Second, the exclusion of right-censored data is an inherent limitation of training the binary classification models presented in this study. Although few participants in CKB were lost to follow-up, and exclusion of these individuals did not lead to a reduction in model performance, care should be taken when developing similar models in other study populations. Finally, the risk equations outlined in the present report were not designed for immediate implementation in clinical practice. Further work is needed to validate and refine the proposed risk prediction models and screening approaches from this study in independent populations in China, and potentially other LMICs, since the CKB cohort may not be representative of the overall Chinese population or other populations. Additional work should also compare the cost and benefits of implementing such approaches over existing care guidelines before implementing them in clinical practice. 

## CONCLUSIONS

Novel risk scores for stroke have been developed using data from a contemporary cohort of 0.5 million Chinese adults. Use of ML techniques improved risk prediction over traditional Cox model approaches, with GBT providing the best discrimination and calibration performance. An ensemble approach was also proposed to screen for individuals at high risk of stroke who may benefit from more intensive treatment. The ensemble approach identified high-risk individuals with marginal improvements to accuracy, specificity, and PPV over either Cox or GBT models alone. By identifying a small portion of individuals who would benefit from ML predictions, our ensemble approach provides an incremental benefit beyond current clinical practice that has potential to translate into important benefits for population health and facilitate the adoption of ML-based risk calculators in clinical practice.

## FUNDING

The baseline survey was supported by the Kadoorie Charitable Foundation, Hong Kong, China and funding for long-term follow-up was provided by the UK Wellcome Trust (202922/Z/16/Z, 104085/Z/14/Z, 088158/Z/09/Z), Chinese National Natural Science Foundation (81390540, 81390541, 81390544), and the National Key Research and Development Program of China (2016YFC0900500, 2016YFC0900501, 2016YFC0900504, 2016YFC1303904). Core funding was provided to the CTSU, University of Oxford, by the British Heart Foundation, the UK Medical Research Council, and Cancer Research UK. MC was supported by a Rhodes Scholarship. BC is supported by a Nuffield Department of Population Health Senior Research Fellowship. The University of Oxford Medical Research Council (MRC) Population Health Research Unit is funded through a strategic partnership between the MRC and the University of Oxford. The research was also supported by the National Institute for Health Research (NIHR) Oxford Biomedical Research Centre (BRC). The views expressed are those of the authors and not necessarily those of the NHS, the NIHR, or the Department of Health.

## AUTHOR CONTRIBUTIONS

Study concept and design: MC, TZ, DC, BC, and RC. Data collection: RC, DB, YC, PP, JL, CY, LY, LL, and ZC. Data analysis and interpretation: MC, TZ, DC, BC, and RC. Drafting of the manuscript: MC, TZ, DC, BC, and RC. Critical revision of the manuscript: all authors. Final approval: all authors.

## DATA AVAILABILITY

The data underlying this article are available in the article and in its online supplementary material. Additional computer code used to estimate the machine learning and other statistical risk prediction models of stroke are available at: https://github.com/ckbiobank/ckb-stroke-risk-models.

## SUPPLEMENTARY MATERIAL

Supplementary material is available at *Journal of the American Medical Informatics Association* online.

## Supplementary Material

ocab068_Supplementary_Data

## Notes

Members of the China Kadoorie Biobank Collaborative Group are provided in the online supplement.
