# Supplementary material for: Stroke risk prediction using machine learning: a prospective cohort study of 0.5 million Chinese adults
Source: J Am Med Inform Assoc. 2021 May 9;28(8):1719–27. doi: 10.1093/jamia/ocab068 (PMC8324240; doi:10.1093/jamia/ocab068)
Supplement: ocab068_Supplementary_Data [file ocab068_supplementary_data.zip › JAMIA - Provided Code to Run Models for External Validation.pdf]

Corresponding code is available at: https://github.com/ckbiobank/ckb-stroke-risk-models

Comparing Model Outputs for New Individuals for External Validation

This notebook allows a user to enter the risk factor data for a new individual and compare the outputs of the following models:

- 2017 Framingham Stroke Risk Profile (FSRP)
- Recalibrated and Refitted FSRP
- China Kadoorie Biobank (CKB) Cox Model
- Logistic Regression (LR)
- Linear Support Vector Machine (SVM)
- Gradient Boosted Tree (GBT)
- Multilayer Perceptron (MLP)

The model outputs include estimates of 9-year stroke risk as well as risk of stroke during years 0-3, 3-6, and 6-9 after risk factor measurements. All models have been trained separately for men and women.

First we import the required dependencies:

In [1]:

```
import warnings
warnings.filterwarnings('ignore')

import pandas as pd
import numpy as np
import matplotlib.pyplot as plt
from pickle import load
from keras.models import load_model
```

```
Using TensorFlow backend.
```

Next, the user is able to enter risk factor data for a new individual. If any data is unavailable (apart from sex and region), the user can enter 'Missing' as a string.

Note: This should be the only cell in which the user modifies the code.

In [2]:

```
#ENTER RISK FACTOR DATA HERE:
#IF DATA UNAVAILABLE, ENTER 'Missing' AND VALUE WILL BE IMPUTED

sex = 'Female' #Set as 'Male' or 'Female'. 'Missing' is not an option for this feature.
age = 43 #Enter age in years
region = 'Haikou' #Set as 'Qingdao','Harbin','Haikou','Suzhou','Liuzhou','Sichuan','Gan
su','Henan','Zhejiang', or 'Hunan'. 'Missing' is not an option for this feature.
sbp_mean = 120 #Systolic blood pressure in mmHg (mean of two measurements)
used_blood_pressure_drugs = 0 #Do you use blood pressure drugs? (0: no, 1: yes)
has_diabetes = 0 #Do you have diabetes? (0: no, 1: yes)
household_size = 4 #How many people live together in your household?
has_health_cover = 1 #Do you have health care coverage? (0: no, 1: yes)
years_since_quitting_smoking = 0 #How many years since quitting smoking? (0 if you curr
ently smoke or never smoked; decimal values allowed)
has_copd = 0 #Has a doctor EVER told you that you had COPD? (0: no, 1: yes)
hypertension_diag = 0 #Has a doctor EVER told you that you had hypertension? (0: no, 1:
yes)
rheum_heart_dis_diag = 0 #Has a doctor EVER told you that you had rheumatic heart disea
se? (0: no, 1: yes)
tb_diag = 0 #Has a doctor EVER told you that you had TB? (0: no, 1: yes)
cirrhosis_hep_diag = 0 #Has a doctor EVER told you that you had cirrhosis/chronic hepat
itis? (0: no, 1: yes)
peptic_ulcer_diag = 0 #Has a doctor EVER told you that you had a peptic ulcer? (0: no,
 1: yes)
gall_diag = 0 #Has a doctor EVER told you that you had a gallstone/gallbladder dis.?
 (0: no, 1: yes)
asthma_diag = 0 #Has a doctor EVER told you that you had asthma? (0: no, 1: yes)
kidney_dis_diag = 0 #Has a doctor EVER told you that you had kidney disease? (0: no, 1:
yes)
fracture_diag = 0 #Has a doctor EVER told you that you had a fracture? (0: no, 1: yes)
rheum_arthritis_diag = 0 #Has a doctor EVER told you that you had rheumatoid arthritis?
(0: no, 1: yes)
neurasthenia_diag = 0 #Has a doctor EVER told you that you had neurasthenia? (0: no, 1:
yes)
head_injury_diag = 0 #Has a doctor EVER told you that you had a head injury? (0: no, 1:
yes)
cancer_diag = 0 #Has a doctor EVER told you that you had cancer? (0: no, 1: yes)
blood_transfusions = 0 #How many blood transfusions have you received? (if none, put 0)
children = 0 #How many children do you have?
siblings = 0 #How many siblings do you have?
mother_still_alive = 1 #Is your mother still alive? (0: no, 1: yes)
father_still_alive = 1 #Is your father still alive? (0: no, 1: yes)
mother_stroke = 0 #Did your mother ever have a stroke? (0: no, 1: yes)
mother_heart_attack = 0 #Did your mother ever have a heart attack? (0: no, 1: yes)
mother_diabetes = 0 #Did your mother ever have diabetes? (0: no, 1: yes)
mother_cancer = 0 #Did your mother ever have cancer? (0: no, 1: yes)
father_stroke = 0 #Did your father ever have a stroke? (0: no, 1: yes)
father_diabetes = 0 #Did your father ever have diabetes? (0: no, 1: yes)
father_heart_attack = 0 #Did your father ever have a heart attack? (0: no, 1: yes)
father_cancer = 0 #Did your father ever have cancer? (0: no, 1: yes)
siblings_stroke = 0 #Did your siblings (incl. half siblings) ever have a stroke? (0: if
no, else record the number with disease)
siblings_diabetes = 0 #Did your siblings (incl. half siblings) ever have diabetes? (0:
 if no, else record the number with disease)
siblings_heart_attack = 0 #Did your siblings (incl. half siblings) ever have a heart at
tack? (0: if no, else record the number with disease)
siblings_cancer = 0 #Did your siblings (incl. half siblings) ever have cancer? (0: if n
o, else record the number with disease)
children_stroke = 0 #Did your children ever have a stroke? (0: if no, else record the n
```

```
umber with disease)
children_heart_attack = 0 #Did your children ever have a heart attack? (0: if no, else
 record the number with disease)
children_diabetes = 0 #Did your children ever have diabetes? (0: if no, else record the
number with disease)
children_cancer = 0 #Did your children ever have cancer? (0: if no, else record the num
ber with disease)
met = 21.3 #Total daily physical activity (Metabolic Equivalent of Task)
met_hours = 6.2 #Total daily hours spent on physical activity
standing_height_cm = 170 #Standing height in cm (without shoes)
sitting_height_cm = 89.2 #Sitting height in cm
waist_cm = 75 #Waist measurement in cm
waist_hip_ratio_percent = 90 #Waist to hip ratio (percent * 100)
weight_kg = 60 #Weight in kg (without shoes, but in light clothing)
bmi_calc = 23.1 #BMI calculated from measured height and weight (in kg/m^2)
fat_percent = 10.2 #Body fat percentage (percent * 100)
dbp_mean = 83 #Diastolic BP in mmHg (mean of two measurements)
heart_rate_mean_10s = 7.3 #Heart rate (bpm/10)
smoking_category_1 = 1 #Does the following characterize your smoking behavior? -- Never
smoker (0: no, 1: yes)
smoking_category_2 = 0 #Does the following characterize your smoking behavior? -- Occas
ional smoker (0: no, 1: yes)
smoking_category_3 = 0 #Does the following characterize your smoking behavior? -- Ex re
gular smoker (0: no, 1: yes)
smoking_category_4 = 0 #Does the following characterize your smoking behavior? -- Regul
ar smoker (0: no, 1: yes)
chd_diag = 0 #Has a doctor EVER told you that you had coronary heart disease? (0: no,
 1: yes)
emph_bronc_diag = 0 #Has a doctor EVER told you that you had emphysema/bronchitis? (0:
 no, 1: yes)
psych_disorder_diag = 0 #Has a doctor EVER told you that you have a psychiatric disorde
r? (0: no, 1: yes)
highest_education_0 = 0 #What is the highest level of school education you ever receive
d? -- No formal school (0: no, 1: yes)
highest_education_1 = 0 #What is the highest level of school education you ever receive
d? -- Primary School (0: no, 1: yes)
highest_education_2 = 0 #What is the highest level of school education you ever receive
d? -- Middle School (0: no, 1: yes)
highest_education_3 = 0 #What is the highest level of school education you ever receive
d? -- High School (0: no, 1: yes)
highest_education_4 = 0 #What is the highest level of school education you ever receive
d? -- Technical school / college (0: no, 1: yes)
highest_education_5 = 1 #What is the highest level of school education you ever receive
d? -- University (0: no, 1: yes)
occupation_0 = 0 #What is your current occupation? -- Agriculture & related workers (0:
no, 1: yes)
occupation_1 = 0 #What is your current occupation? -- Factory worker (0: no, 1: yes)
occupation_2 = 0 #What is your current occupation? -- Administrator / manager (0: no,
 1: yes)
occupation_3 = 1 #What is your current occupation? -- Professional / technical (0: no,
 1: yes)
occupation_4 = 0 #What is your current occupation? -- Sales & service workers (0: no,
 1: yes)
occupation_5 = 0 #What is your current occupation? -- Retired (0: no, 1: yes)
occupation_6 = 0 #What is your current occupation? -- House wife / husband (0: no, 1: y
es)
occupation_7 = 0 #What is your current occupation? -- Self-employed (0: no, 1: yes)
occupation_8 = 0 #What is your current occupation? -- Unemployed (0: no, 1: yes)
occupation_9 = 0 #What is your current occupation? -- Other or not stated (0: no, 1: ye
s)
household_income_0 = 0 #What is the total income last year in your household? -- <2,500
```

```
yuan (0: no, 1: yes)
household_income_1 = 0 #What is the total income last year in your household? -- 2,500-
4,999 yuan (0: no, 1: yes)
household_income_2 = 0 #What is the total income last year in your household? -- 5,000-
9,999 yuan (0: no, 1: yes)
household_income_3 = 0 #What is the total income last year in your household? -- 10,000
-19,999 yuan (0: no, 1: yes)
household_income_4 = 0 #What is the total income last year in your household? -- 20,000
-34,999 yuan (0: no, 1: yes)
household_income_5 = 1 #What is the total income last year in your household? -- >=35,0
00 yuan (0: no, 1: yes)
alcohol_category_1 = 0 #Does the following characterize your alcohol intake? -- Never r
egular (0: no; 1: yes)
alcohol_category_2 = 0 #Does the following characterize your alcohol intake? -- Ex-regu
lar (0: no; 1: yes)
alcohol_category_3 = 0 #Does the following characterize your alcohol intake? -- Occasio
nal (0: no; 1: yes)
alcohol_category_4 = 0 #Does the following characterize your alcohol intake? -- Monthly
(0: no; 1: yes)
alcohol_category_5 = 0 #Does the following characterize your alcohol intake? -- Reduced
intake (0: no; 1: yes)
alcohol_category_6 = 1 #Does the following characterize your alcohol intake? -- Weekly
 (0: no; 1: yes)
smoking_now_0 = 1 #Does the following characterize your smoking behavior? -- Do not smo
ke now (0: no; 1: yes)
smoking_now_1 = 0 #Does the following characterize your smoking behavior? -- Only occas
ionally (0: no; 1: yes)
smoking_now_2 = 0 #Does the following characterize your smoking behavior? -- Yes, on mo
st days (0: no; 1: yes)
smoking_now_3 = 0 #Does the following characterize your smoking behavior? -- Yes, daily
or almost every day (0: no; 1: yes)
self_rated_health_0 = 1 #How would you rate your current general health status? -- Exce
llent (0: no, 1: yes)
self_rated_health_1 = 0 #How would you rate your current general health status? -- Good
(0: no, 1: yes)
self_rated_health_2 = 0 #How would you rate your current general health status? -- Fair
(0: no, 1: yes)
self_rated_health_3 = 0 #How would you rate your current general health status? -- Poor
(0: no, 1: yes)
comparative_health_0 = 1 #How would rate your current general health status compared to
someone of your own age? -- Better (0: no, 1: yes)
comparative_health_1 = 0 #How would rate your current general health status compared to
someone of your own age? -- About the same (0: no, 1: yes)
comparative_health_2 = 0 #How would rate your current general health status compared to
someone of your own age? -- Worse (0: no, 1: yes)
comparative_health_3 = 0 #How would rate your current general health status compared to
someone of your own age? -- Don't know (0: no, 1: yes)
diet_freq_rice_0 = 0 #During the past 12 months, about how often did you eat rice? -- D
aily (0: no, 1: yes)
diet_freq_rice_1 = 0 #During the past 12 months, about how often did you eat rice? -- 4
-6 days per week (0: no, 1: yes)
diet_freq_rice_2 = 1 #During the past 12 months, about how often did you eat rice? -- 1
-3 days per week (0: no, 1: yes)
diet_freq_rice_3 = 0 #During the past 12 months, about how often did you eat rice?  --
 Monthly (0: no, 1: yes)
diet_freq_rice_4 = 0 #During the past 12 months, about how often did you eat rice? -- N
ever/rarely (0: no, 1:yes)
diet_freq_wheat_0 = 0 #During the past 12 months, about how often did you eat wheat? --
Daily (0: no, 1: yes)
diet_freq_wheat_1 = 1 #During the past 12 months, about how often did you eat wheat? --
4-6 days per week (0: no, 1: yes)
```

```
diet_freq_wheat_2 = 0 #During the past 12 months, about how often did you eat wheat? -- 1-3 days per week (0: no, 1: yes)
diet_freq_wheat_3 = 0 #During the past 12 months, about how often did you eat wheat?  -- Monthly (0: no, 1: yes)
diet_freq_wheat_4 = 0 #During the past 12 months, about how often did you eat wheat? -- Never/rarely (0: no, 1:yes)
diet_freq_other_staple_0 = 0 #During the past 12 months, about how often did you eat staple foods other than rice and wheat (corn, millet etc.)? -- Daily (0: no, 1: yes)
diet_freq_other_staple_1 = 0 #During the past 12 months, about how often did you eat staple foods other than rice and wheat (corn, millet etc.)? -- 4-6 days per week (0: no, 1: yes)
diet_freq_other_staple_2 = 0 #During the past 12 months, about how often did you eat staple foods other than rice and wheat (corn, millet etc.)? -- 1-3 days per week (0: no, 1: yes)
diet_freq_other_staple_3 = 1 #During the past 12 months, about how often did you eat staple foods other than rice and wheat (corn, millet etc.)?  -- Monthly (0: no, 1: yes)
diet_freq_other_staple_4 = 0 #During the past 12 months, about how often did you eat staple foods other than rice and wheat (corn, millet etc.)? -- Never/rarely (0: no, 1:yes)
bowel_movement_freq_0 = 0 #About how often do you have bowel movements each week? -- More than once on most days (0: no, 1:yes)
bowel_movement_freq_1 = 1 #About how often do you have bowel movements each week? -- About daily (0: no, 1:yes)
bowel_movement_freq_2 = 0 #About how often do you have bowel movements each week? -- Once every 2-3 days (0: no, 1:yes)
bowel_movement_freq_3 = 0 #About how often do you have bowel movements each week? -- Less than 3 times a week (0: no, 1:yes)
gum_bleed_freq_0 = 1 #How often do your gums bleed when you brush your teeth? -- Occasionally, rarely or never (0: no, 1:yes)
gum_bleed_freq_1 = 0 #How often do your gums bleed when you brush your teeth? -- Sometimes (0: no, 1:yes)
gum_bleed_freq_2 = 0 #How often do your gums bleed when you brush your teeth? -- Always (0: no, 1:yes)
gum_bleed_freq_3 = 0 #How often do your gums bleed when you brush your teeth? -- Brush teeth rarely or never (0: no, 1:yes)
missing_mother_history = 0 #Are you unable to answer if your mother had any of the following: stroke, heart attack, diabetes, cancer? (0: no, 1: yes)
missing_father_history = 0 #Are you unable to answer if your father had any of the following: stroke, heart attack, diabetes, cancer? (0: no, 1: yes)
missing_siblings_history = 0 #Are you unable to answer if your siblings had any of the following: stroke, heart attack, diabetes, cancer? (0: no, 1: yes)
missing_children_history = 0 #Are you unable to answer if your children had any of the following: stroke, heart attack, diabetes, cancer? (0: no, 1: yes)
```

In the following cell, backend processing is done to:

- **Calculate derived risk factors from the user input**
- **Prepare the input vector to the models**
- **Impute missing data with mean values from the CKB training set**

In [3]:

```
#CALCULATE DERIVED RISK FACTORS:

#region_is_urban
if region in ['Qingdao','Harbin','Haikou','Suzhou','Liuzhou']:
    region_is_urban = 1
else:
```

```
    region_is_urban = 0

#age_at_study_date
if (age == 'Missing'):
    age_at_study_date = 'Missing'
else:
    age_at_study_date = age/10


#over_65
if (age == 'Missing'):
    over_65 = 'Missing'
elif age >= 65:
    over_65 = 1
else:
    over_65 = 0

#diab_under_65
if (over_65 == 'Missing')|(has_diabetes == 'Missing'):
    diab_under_65 = 'Missing'
elif (over_65 == 0) & (has_diabetes == 1):
    diab_under_65 = 1
else:
    diab_under_65 = 0

#diab_over_65
if (over_65 == 'Missing')|(has_diabetes == 'Missing'):
    diab_over_65 = 'Missing'
elif (over_65 == 1) & (has_diabetes == 1):
    diab_over_65 = 1
else:
    diab_over_65 = 0

#sbp_noHRX
if (sbp_mean == 'Missing')|(used_blood_pressure_drugs == 'Missing'):
    sbp_noHRX = 'Missing'
else:
    sbp_noHRX = ((sbp_mean-120)*(1-used_blood_pressure_drugs))/10

#sbp_HRX
if (sbp_mean == 'Missing')|(used_blood_pressure_drugs == 'Missing'):
    sbp_HRX = 'Missing'
else:
    sbp_HRX = ((sbp_mean-120)*(used_blood_pressure_drugs))/10

#sbp_mean
if (sbp_mean == 'Missing'):
    sbp_mean = 'Missing'
else:
    sbp_mean = sbp_mean/10

#######################################################################################
###################
#CREATE INPUT VECTOR

column_names = ['region','region_is_urban','age_at_study_date','sbp_mean','used_blood_p
ressure_drugs','has_diabetes',
                'household_size','has_health_cover','years_since_quitting_smoking','has
_copd','hypertension_diag',
                'rheum_heart_dis_diag','tb_diag','cirrhosis_hep_diag','peptic_ulcer_dia
g','gall_diag','asthma_diag',
```

```
                  'kidney_dis_diag','fracture_diag','rheum_arthritis_diag','neurasthenia_
diag','head_injury_diag',
                  'cancer_diag','blood_transfusions','children','siblings','mother_still_
alive','father_still_alive',
                  'mother_stroke','mother_heart_attack','mother_diabetes','mother_cancer'
,'father_stroke','father_diabetes',
                  'father_heart_attack','father_cancer','siblings_stroke','siblings_diabe
tes','siblings_heart_attack',
                  'siblings_cancer','children_stroke','children_heart_attack','children_d
iabetes','children_cancer','met',
                  'met_hours','standing_height_cm','sitting_height_cm','waist_cm','waist_
hip_ratio_percent','weight_kg',
                  'bmi_calc','fat_percent','dbp_mean','heart_rate_mean_10s','over_65','sm
oking_category_1',
                  'smoking_category_2','smoking_category_3','smoking_category_4','diab_un
der_65','diab_over_65','sbp_noHRX',
                  'sbp_HRX','chd_diag','emph_bronc_diag','psych_disorder_diag','highest_e
ducation_0','highest_education_1',
                  'highest_education_2','highest_education_3','highest_education_4','high
est_education_5','occupation_0',
                  'occupation_1','occupation_2','occupation_3','occupation_4','occupation
_5','occupation_6','occupation_7',
                  'occupation_8','occupation_9','household_income_0','household_income_1'
,'household_income_2',
                  'household_income_3','household_income_4','household_income_5','alcohol
_category_1','alcohol_category_2',
                  'alcohol_category_3','alcohol_category_4','alcohol_category_5','alcohol
_category_6','smoking_now_0',
                  'smoking_now_1','smoking_now_2','smoking_now_3','self_rated_health_0',
'self_rated_health_1',
                  'self_rated_health_2','self_rated_health_3','comparative_health_0','com
parative_health_1',
                  'comparative_health_2','comparative_health_3','diet_freq_rice_0','diet_
freq_rice_1','diet_freq_rice_2',
                  'diet_freq_rice_3','diet_freq_rice_4','diet_freq_wheat_0','diet_freq_wh
eat_1','diet_freq_wheat_2',
                  'diet_freq_wheat_3','diet_freq_wheat_4','diet_freq_other_staple_0','die
t_freq_other_staple_1',
                  'diet_freq_other_staple_2','diet_freq_other_staple_3','diet_freq_other_
staple_4','bowel_movement_freq_0',
                  'bowel_movement_freq_1','bowel_movement_freq_2','bowel_movement_freq_3'
,'gum_bleed_freq_0',
                  'gum_bleed_freq_1','gum_bleed_freq_2','gum_bleed_freq_3','missing_mothe
r_history','missing_father_history',
                  'missing_siblings_history','missing_children_history']

risk_factor_df = pd.DataFrame(columns = column_names)
risk_factor_df = risk_factor_df.append({
    'region':region,'region_is_urban':region_is_urban,'age_at_study_date':age_at_study_
date,'sbp_mean':sbp_mean,
    'used_blood_pressure_drugs':used_blood_pressure_drugs,'has_diabetes':has_diabetes,
'household_size':household_size,
    'has_health_cover':has_health_cover,'years_since_quitting_smoking':years_since_quit
ting_smoking,'has_copd':has_copd,
    'hypertension_diag':hypertension_diag,'rheum_heart_dis_diag':rheum_heart_dis_diag,
'tb_diag':tb_diag,
    'cirrhosis_hep_diag':cirrhosis_hep_diag,'peptic_ulcer_diag':peptic_ulcer_diag,'gall
_diag':gall_diag,
    'asthma_diag':asthma_diag,'kidney_dis_diag':kidney_dis_diag,'fracture_diag':fractur
e_diag,
    'rheum_arthritis_diag':rheum_arthritis_diag,'neurasthenia_diag':neurasthenia_diag,
```

```
'head_injury_diag':head_injury_diag,
    'cancer_diag':cancer_diag,'blood_transfusions':blood_transfusions,'children':childr
en,'siblings':siblings,
    'mother_still_alive':mother_still_alive,'father_still_alive':father_still_alive,'mo
ther_stroke':mother_stroke,
    'mother_heart_attack':mother_heart_attack,'mother_diabetes':mother_diabetes,'mother
_cancer':mother_cancer,
    'father_stroke':father_stroke,'father_diabetes':father_diabetes,'father_heart_attac
k':father_heart_attack,
    'father_cancer':father_cancer,'siblings_stroke':siblings_stroke,'siblings_diabetes'
:siblings_diabetes,
    'siblings_heart_attack':siblings_heart_attack,'siblings_cancer':siblings_cancer,'ch
ildren_stroke':children_stroke,
    'children_heart_attack':children_heart_attack,'children_diabetes':children_diabetes
,'children_cancer':children_cancer,
    'met':met,'met_hours':met_hours,'standing_height_cm':standing_height_cm,'sitting_he
ight_cm':sitting_height_cm,
    'waist_cm':waist_cm,'waist_hip_ratio_percent':waist_hip_ratio_percent,'weight_kg':w
eight_kg,'bmi_calc':bmi_calc,
    'fat_percent':fat_percent,'dbp_mean':dbp_mean,'heart_rate_mean_10s':heart_rate_mean
_10s,'over_65':over_65,
    'smoking_category_1':smoking_category_1,'smoking_category_2':smoking_category_2,'sm
oking_category_3':smoking_category_3,
    'smoking_category_4':smoking_category_4,'diab_under_65':diab_under_65,'diab_over_6
5':diab_over_65,'sbp_noHRX':sbp_noHRX,
    'sbp_HRX':sbp_HRX,'chd_diag':chd_diag,'emph_bronc_diag':emph_bronc_diag,'psych_diso
rder_diag':psych_disorder_diag,
    'highest_education_0':highest_education_0,'highest_education_1':highest_education_1
,'highest_education_2':highest_education_2,
    'highest_education_3':highest_education_3,'highest_education_4':highest_education_4
,'highest_education_5':highest_education_5,
    'occupation_0':occupation_0,'occupation_1':occupation_1,'occupation_2':occupation_2
,'occupation_3':occupation_3,
    'occupation_4':occupation_4,'occupation_5':occupation_5,'occupation_6':occupation_6
,'occupation_7':occupation_7,
    'occupation_8':occupation_8,'occupation_9':occupation_9,'household_income_0':househ
old_income_0,
    'household_income_1':household_income_1,'household_income_2':household_income_2,'ho
usehold_income_3':household_income_3,
    'household_income_4':household_income_4,'household_income_5':household_income_5,'al
cohol_category_1':alcohol_category_1,
    'alcohol_category_2':alcohol_category_2,'alcohol_category_3':alcohol_category_3,'al
cohol_category_4':alcohol_category_4,
    'alcohol_category_5':alcohol_category_5,'alcohol_category_6':alcohol_category_6,'sm
oking_now_0':smoking_now_0,
    'smoking_now_1':smoking_now_1,'smoking_now_2':smoking_now_2,'smoking_now_3':smoking
_now_3,'self_rated_health_0':self_rated_health_0,
    'self_rated_health_1':self_rated_health_1,'self_rated_health_2':self_rated_health_2
,'self_rated_health_3':self_rated_health_3,
    'comparative_health_0':comparative_health_0,'comparative_health_1':comparative_heal
th_1,'comparative_health_2':comparative_health_2,
    'comparative_health_3':comparative_health_3,'diet_freq_rice_0':diet_freq_rice_0,'di
et_freq_rice_1':diet_freq_rice_1,
    'diet_freq_rice_2':diet_freq_rice_2,'diet_freq_rice_3':diet_freq_rice_3,'diet_freq_
rice_4':diet_freq_rice_4,
    'diet_freq_wheat_0':diet_freq_wheat_0,'diet_freq_wheat_1':diet_freq_wheat_1,'diet_f
req_wheat_2':diet_freq_wheat_2,
    'diet_freq_wheat_3':diet_freq_wheat_3,'diet_freq_wheat_4':diet_freq_wheat_4,'diet_f
req_other_staple_0':diet_freq_other_staple_0,
    'diet_freq_other_staple_1':diet_freq_other_staple_1,'diet_freq_other_staple_2':diet
_freq_other_staple_2,
```

```
    'diet_freq_other_staple_3':diet_freq_other_staple_3,'diet_freq_other_staple_4':diet
_freq_other_staple_4,
    'bowel_movement_freq_0':bowel_movement_freq_0,'bowel_movement_freq_1':bowel_movemen
t_freq_1,
    'bowel_movement_freq_2':bowel_movement_freq_2,'bowel_movement_freq_3':bowel_movemen
t_freq_3,'gum_bleed_freq_0':gum_bleed_freq_0,
    'gum_bleed_freq_1':gum_bleed_freq_1,'gum_bleed_freq_2':gum_bleed_freq_2,'gum_bleed_
freq_3':gum_bleed_freq_3,
    'missing_mother_history':missing_mother_history,'missing_father_history':missing_fa
ther_history,
    'missing_siblings_history':missing_siblings_history,'missing_children_history':miss
ing_children_history
},ignore_index = True)

#If any values are 'Missing', replace with CKB mean value
male_mean_value_df = pd.DataFrame(columns = column_names)
male_mean_value_df.loc[len(male_mean_value_df)]=['NA',0.430950498,5.263505828,13.254029
27,0.099336382,0.05290032,
                                                 3.80744765,0.8484911,1.394061345,0.087
129938,0.10272324,0.000957031,
                                                 0.019891345,0.017249481,0.053169664,0.
038447432,0.005650495,0.012424211,
                                                 0.088350583,0.014189274,0.007203521,0.
016550333,0.00450435,0.054098041,
                                                 2.012664902,3.339219251,0.567616821,0.
711515318,0.080231331,0.013006609,
                                                 0.031842383,0.060271033,0.09662493,0.0
16860996,0.015655409,0.094529505,
                                                 0.030217181,0.030068073,0.005339191,0.
040362216,0.000458458,0.000252152,
                                                 0.001318067,0.00189687,22.29973685,6.3
55241264,165.2396005,88.35389059,
                                                 81.93837121,90.29892606,64.11819114,2
3.40745686,21.94969035,79.09611858,
                                                 7.769930887,0.163474653,0.143061812,0.
113027083,0.12850004,0.615411065,
                                                 0.038814198,0.014086121,0.966956928,0.
287072345,0.024899999,0.030882875,
                                                 0.002699171,0.088585543,0.333654254,0.
325018052,0.175016333,0.044613692,
                                                 0.033112127,0.438778668,0.194237183,0.
034504693,0.037524785,0.049519192,
                                                 0.139193572,0.02617222,0.035799837,0.0
27324095,0.016945753,0.02885993,
                                                 0.063169778,0.168191039,0.282152231,0.
25415191,0.203475111,0.201824663,
                                                 0.034923036,0.316370388,0.063525083,0.
047714014,0.335642815,0.322496533,
                                                 0.094877878,0.016951484,0.565674105,0.
203085422,0.296714002,0.417844331,
                                                 0.082356245,0.207291774,0.635371179,0.
130224988,0.027112059,0.686391821,
                                                 0.019719424,0.159319878,0.097519742,0.
037049135,0.415832846,0.082436475,
                                                 0.240197595,0.181692627,0.079840457,0.
135204988,0.007341058,0.114774954,
                                                 0.427546448,0.315132552,0.126608901,0.
790100746,0.064229963,0.01906039,
                                                 0.654981719,0.191790164,0.037415902,0.
115812216,0.001157606,0.001770794,0.000727802,0]
```

```
female_mean_value_df = pd.DataFrame(columns = column_names)
female_mean_value_df.loc[len(female_mean_value_df)]=['NA',0.444027175,5.132173889,12.96
632725,0.114239102,0.059617916,
                                                    3.79464152,0.802577966,0.102426382
,0.06137544,0.112134801,0.002313155,
                                                    0.011163828,0.008405381,0.02917648
5,0.075124327,0.005260752,
                                                    0.015912297,0.055732446,0.02476297
1,0.013433636,0.00703798,0.005252871,
                                                    0.06631306,2.127101345,3.536107571
,0.533984064,0.686364604,0.082093177,
                                                    0.013713126,0.034616463,0.05738743
8,0.093056702,0.018068457,0.015369844,
                                                    0.090314627,0.031378846,0.03559267
9,0.005878848,0.043250964,0.000634443,
                                                    0.000267963,0.001501383,0.00248654
3,20.54483875,6.430382321,154.1575952,
                                                    83.16809699,78.98875027,86.6218208
9,56.67632889,23.80129608,32.06847923,
                                                    76.70663328,7.965437056,0.12561572
5,0.94980021,0.018485534,0.00846055,
                                                    0.023253706,0.043276089,0.01634182
7,0.614425494,0.351901752,0.030496599,
                                                    0.022272487,0.004515971,0.25234665
,0.314222551,0.254159344,0.134840759,
                                                    0.029105554,0.015325142,0.40839986
4,0.107839506,0.013319357,0.026516555,
                                                    0.050227375,0.174566333,0.15565521
,0.021957236,0.02593334,0.015585224,
                                                    0.030390202,0.070639881,0.19593247
3,0.295173506,0.242881237,0.164982701,
                                                    0.635400329,0.004334702,0.32098074
6,0.014028672,0.004393812,0.020861739,
                                                    0.96801384,0.010328413,0.001083676
,0.020574072,0.161641039,0.27537574,
                                                    0.45320098,0.10978224,0.167768732,
0.626293514,0.172639361,0.033298393,
                                                    0.703924088,0.020507081,0.15318837
,0.093156688,0.029223773,0.383696003,
                                                    0.066104206,0.226677333,0.20981534
2,0.113707116,0.125757588,0.009721555,
                                                    0.135049613,0.45881639,0.270654855
,0.075427756,0.747641528,0.116071499,
                                                    0.060859217,0.623428671,0.23385717
6,0.064181175,0.078532979,0.001990022,
                                                    0.00265205,0.001241301,0]

for column in risk_factor_df:
    if (sex == 'Male'):
        risk_factor_df.loc[risk_factor_df[column] == 'Missing', column] = male_mean_val
ue_df[column][0]
    else:
        risk_factor_df.loc[risk_factor_df[column] == 'Missing', column] = female_mean_v
alue_df[column][0]
```

In the following cell, we calculate stroke risk estimates using the FSRP, as presented in Dufouil et al., 2017 with the exception of atrial fibrillation, which was not recorded in CKB.

(Link to paper: [https://doi.org/10.1161/CIRCULATIONAHA.115.021275 (https://doi.org/10.1161/CIRCULATIONAHA.115.021275)](https://doi.org/10.1161/CIRCULATIONAHA.115.021275))

In [4]:

```
#FSRP (without atrial fibrillation) Output

FSRP_risk_factor_df = risk_factor_df.copy()
FSRP_risk_factor_df['smoking_now'] = np.where(risk_factor_df['smoking_now_0']==1, 0, 1)

if (sex == 'Male'):
    #Vector format: [Age/10,smoking,CVD,65+,Diab_65-,Diab_65+,BP_drugs,sbp_noHRX,sbp_HR
X]
    male_coeffs = np.array([0.49716,0.47254,0.45341,0.45426,1.35304,0.34385,0.82598,0.2
7323,0.09793])
    male_means = np.array([6.6753588,0.124400,0.1798341,0.5229158,0.058490,0.092100,0.4
212134,0.6656045,0.8152772])
    S_3_male = 0.98919
    S_6_male = 0.97661
    S_9_male = 0.95509

    M_male = male_coeffs.dot(male_means)
    x_male = FSRP_risk_factor_df[['age_at_study_date','smoking_now','chd_diag','over_6
5','diab_under_65','diab_over_65','used_blood_pressure_drugs','sbp_noHRX','sbp_HRX']].v
alues
    L_male = x_male.dot(male_coeffs)
    A_male = L_male-M_male
    B_male = np.exp(A_male.astype(float))

    p_male_3 = 1-(S_3_male**B_male)
    p_male_6 = 1-(S_6_male**B_male)
    p_male_9 = 1-(S_9_male**B_male)


    FSRP_9yr_risk = p_male_9
    FSRP_0_3yr_risk = p_male_3
    FSRP_3_6yr_risk = p_male_6-p_male_3
    FSRP_6_9yr_risk = p_male_9-p_male_6

else:
    #Vector format: [Age/10,smoking,CVD,65+,Diab_65-,Diab_65+,BP_drugs,sbp_noHRX,sbp_HR
X]
    female_coeffs = np.array([0.87938,0.51127,-0.03035,0.39796,1.07111,0.06565,0.13085,
0.11303,0.17234])
    female_means = np.array([6.7870368,0.1384394,0.1006832,0.568141,0.0320029,0.0564545
,0.396980,0.5706221,0.9388709])
    S_3_female = 0.99424
    S_6_female = 0.98327
    S_9_female = 0.96581

    #Female Model
    M_female = female_coeffs.dot(female_means)
    x_female = FSRP_risk_factor_df[['age_at_study_date','smoking_now','chd_diag','over_
65','diab_under_65','diab_over_65','used_blood_pressure_drugs','sbp_noHRX','sbp_HRX']].
values
    L_female = x_female.dot(female_coeffs)
    A_female = L_female-M_female
```

```
    B_female = np.exp(A_female.astype(float))
    p_female = 1-(S_9_female**B_female)

    p_female_3 = 1-(S_3_female**B_female)
    p_female_6 = 1-(S_6_female**B_female)
    p_female_9 = 1-(S_9_female**B_female)

    FSRP_9yr_risk = p_female
    FSRP_0_3yr_risk = p_female_3
    FSRP_3_6yr_risk = p_female_6-p_female_3
    FSRP_6_9yr_risk = p_female_9-p_female_6
```

In the following cell, we calculate stroke risk estimates using a recalibrated and refitted FSRP trained using CKB data. The risk factor coefficients and baseline survival functions for each region are included below.

In [5]:

```
#Recalibrated and Refitted FSRP (without atrial fibrillation) Output

FSRP_risk_factor_df = risk_factor_df.copy()
FSRP_risk_factor_df['smoking_now'] = np.where(risk_factor_df['smoking_now_0']==1, 0, 1)

region = FSRP_risk_factor_df['region'][0]

if (sex == 'Male'):
    #Vector format: [Age/10,smoking,CVD,65+,Diab_65-,Diab_65+,BP_drugs,sbp_noHRX,sbp_HR
X]
    male_coeffs = np.array([0.69199696,0.16897723,0.16314926,-0.13967709,0.50723661,0.2
4428757,0.70359651,0.19377743,0.0894212])

    male_means = np.array([5.26350583, 0.67750347, 0.0249    , 0.16347465, 0.0388142 ,
        0.01408612, 0.09933638, 0.96695693, 0.28707234])

    Survival_retrained_male = {'Qingdao': np.array([[1.        , 0.9975566 , 0.99485713
, 0.99181601, 0.98876377,
         0.98474063, 0.97987223, 0.97453526, 0.96801066, 0.96078314]]),
 'Harbin': np.array([[1.        , 0.99261516, 0.98163398, 0.96882976, 0.95334651,
         0.93584728, 0.91634063, 0.89663451, 0.87218306, 0.84916639]]),
 'Haikou': np.array([[1.        , 0.99456542, 0.9885399 , 0.98123277, 0.97217516,
         0.96101014, 0.94541524, 0.9316325 , 0.91587629, 0.90047497]]),
 'Suzhou': np.array([[1.        , 0.99890109, 0.99745648, 0.99545061, 0.99262683,
         0.98880074, 0.98445847, 0.98007265, 0.97414271, 0.96899247]]),
 'Liuzhou': np.array([[1.        , 0.99603467, 0.98957545, 0.98229644, 0.97398881,
         0.966129  , 0.95894933, 0.95064513, 0.94155596, 0.93235449]]),
 'Sichuan': np.array([[1.        , 0.99770262, 0.99546927, 0.99184998, 0.98796332,
         0.98387353, 0.97896717, 0.97247347, 0.96595605, 0.95617512]]),
 'Gansu': np.array([[1.        , 0.99691333, 0.99215479, 0.98719154, 0.98158817,
         0.9739304 , 0.96566313, 0.95598842, 0.94638019, 0.93507836]]),
 'Henan': np.array([[1.        , 0.99426241, 0.98800017, 0.97961605, 0.96963398,
         0.95826425, 0.94623292, 0.93148483, 0.91489355, 0.89904672]]),
 'Zhejiang': np.array([[1.        , 0.99769469, 0.9940403 , 0.9898103 , 0.9866152 ,
         0.98269232, 0.97958699, 0.97534397, 0.97145794, 0.96667647]]),
 'Hunan': np.array([[1.        , 0.99666281, 0.99114382, 0.98358032, 0.97585018,
         0.96801455, 0.95991193, 0.95107662, 0.9404145 , 0.92870802]])}

    S_3_male = Survival_retrained_male[region][0,3]
    S_6_male = Survival_retrained_male[region][0,6]
    S_9_male = Survival_retrained_male[region][0,9]
```

```
    M_male = male_coeffs.dot(male_means)
    x_male = FSRP_risk_factor_df[['age_at_study_date','smoking_now','chd_diag','over_6
5','diab_under_65','diab_over_65','used_blood_pressure_drugs','sbp_noHRX','sbp_HRX']].v
alues
    L_male = x_male.dot(male_coeffs)
    A_male = L_male-M_male
    B_male = np.exp(A_male.astype(float))

    p_male_3 = 1-(S_3_male**B_male)
    p_male_6 = 1-(S_6_male**B_male)
    p_male_9 = 1-(S_9_male**B_male)


    Recalibrated_Refitted_FSRP_9yr_risk = p_male_9
    Recalibrated_Refitted_FSRP_0_3yr_risk = p_male_3
    Recalibrated_Refitted_FSRP_3_6yr_risk = p_male_6-p_male_3
    Recalibrated_Refitted_FSRP_6_9yr_risk = p_male_9-p_male_6

else:
    #Vector format: [Age/10,smoking,CVD,65+,Diab_65-,Diab_65+,BP_drugs,sbp_noHRX,sbp_HR
X]
    female_coeffs = np.array([0.69692596,0.16086501,0.25319997,-0.24213473,0.43148075,
0.2379541 ,0.56714713,0.142126,0.07889848])

    female_means = np.array([5.13217389, 0.03198616, 0.0304966 , 0.12561572, 0.04327609
,
        0.01634183, 0.1142391 , 0.61442549, 0.35190175])

    Survival_retrained_female = {'Qingdao': np.array([[1.        , 0.99805202, 0.995911
33, 0.99328068, 0.99068026,
         0.98749526, 0.9846341 , 0.98054427, 0.97651665, 0.97163017]]),
 'Harbin': np.array([[1.        , 0.99553194, 0.98803072, 0.97826353, 0.96558413,
         0.95058084, 0.93071238, 0.91002659, 0.88601203, 0.86315973]]),
 'Haikou': np.array([[1.        , 0.99581926, 0.99175812, 0.9851365 , 0.97556374,
         0.96315349, 0.94777552, 0.93494663, 0.9204787 , 0.90653359]]),
 'Suzhou': np.array([[1.        , 0.99941436, 0.99854403, 0.99695708, 0.99523574,
         0.99280071, 0.98994635, 0.98657415, 0.9828433 , 0.97823997]]),
 'Liuzhou': np.array([[1.        , 0.99708402, 0.99353681, 0.98918471, 0.9846092 ,
         0.97999273, 0.9740458 , 0.96775772, 0.96192713, 0.95600553]]),
 'Sichuan': np.array([[1.        , 0.99887589, 0.99744958, 0.99520182, 0.99243524,
         0.98918469, 0.98564964, 0.98090228, 0.97447887, 0.96606237]]),
 'Gansu': np.array([[1.        , 0.99791181, 0.99455501, 0.99006496, 0.98531873,
         0.98000519, 0.97261027, 0.96423064, 0.95366297, 0.94178573]]),
 'Henan': np.array([[1.        , 0.99593043, 0.99115281, 0.98466609, 0.97805243,
         0.96904611, 0.95764649, 0.94653529, 0.93304965, 0.9200178 ]]),
 'Zhejiang': np.array([[1.        , 0.99841621, 0.99627358, 0.99351813, 0.99087261,
         0.98840672, 0.98590643, 0.98289719, 0.98029609, 0.9770322 ]]),
 'Hunan': np.array([[1.        , 0.99697665, 0.9930153 , 0.98766481, 0.9813037 ,
         0.97513623, 0.96824168, 0.96199054, 0.95526356, 0.94736965]])}

    S_3_female = Survival_retrained_female[region][0,3]
    S_6_female = Survival_retrained_female[region][0,6]
    S_9_female = Survival_retrained_female[region][0,9]

    #Female Model
    M_female = female_coeffs.dot(female_means)
    x_female = FSRP_risk_factor_df[['age_at_study_date','smoking_now','chd_diag','over_
65','diab_under_65','diab_over_65','used_blood_pressure_drugs','sbp_noHRX','sbp_HRX']].
values
    L_female = x_female.dot(female_coeffs)
```

```
    A_female = L_female-M_female
    B_female = np.exp(A_female.astype(float))
    p_female = 1-(S_9_female**B_female)

    p_female_3 = 1-(S_3_female**B_female)
    p_female_6 = 1-(S_6_female**B_female)
    p_female_9 = 1-(S_9_female**B_female)

    Recalibrated_Refitted_FSRP_9yr_risk = p_female_9
    Recalibrated_Refitted_FSRP_0_3yr_risk = p_female_3
    Recalibrated_Refitted_FSRP_3_6yr_risk = p_female_6-p_female_3
    Recalibrated_Refitted_FSRP_6_9yr_risk = p_female_9-p_female_6
```

In the following cell, we calculate stroke risk estimates using a Cox model with additional risk factors recorded in CKB. The risk factor coefficients and baseline survival functions for each region are included below.

In [6]:

```
#CKB Cox Model Output

region = risk_factor_df['region'][0]

if (sex == 'Male'):
    #Vector format: [Age/10,smoking,CVD,65+,Diab_65-,Diab_65+,BP_drugs,sbp_noHRX,sbp_HR
X]
    male_coeffs = np.array([-9.60205232e-11,  5.93647219e-01,  1.89907663e-02,  4.78775
851e-01,
        2.31580942e-01,  5.84662635e-04, -1.41382043e-03,  2.09588829e-02,
        1.87813535e-01,  1.12818331e-02,  1.06545281e-01, -5.50350769e-03,
        1.42200357e-01,  1.42195006e-01,  1.08736594e-01,  8.29421922e-02,
        1.10768835e-01, -9.28134249e-02,  6.48088373e-02,  7.48465397e-02,
        7.52334772e-02, -2.92496402e-03, -3.92067630e-03, -1.85462051e-02,
        1.23438470e-02, -1.03124067e-02,  1.74392451e-02,  4.79191309e-02,
        2.19935104e-01,  8.84805162e-02,  6.53596143e-02, -1.02742782e-01,
        8.19074951e-02,  2.37159825e-02, -5.13178995e-03, -9.13949151e-02,
       -7.45700188e-02, -7.69461793e-03, -3.63447385e-02,  7.14993360e-02,
       -5.43368358e-02,  1.01988778e-02, -3.14220487e-03,  6.91341956e-02,
        1.73650077e-01, -5.04167875e-02,  7.33634333e-02,  1.73307332e-01,
       -5.61270164e-03,  5.72816319e-02,  1.21471614e-01, -6.66525290e-02,
        1.76584561e-01,  1.33263208e-01,  8.34471387e-03, -1.04073933e-02,
       -2.22028040e-02, -5.74010217e-03, -5.47385638e-02, -1.18155826e-01,
       -6.68293887e-02,  5.59397072e-03,  2.06442770e-02,  2.50084088e-01,
       -5.31043129e-02,  3.66644976e-02])

    male_means = np.array([4.30950498e-01, 5.26350583e+00, 1.32540293e+01, 9.93363821e-
02,
        5.29003198e-02, 3.80744765e+00, 1.39406135e+00, 8.71299385e-02,
        1.02723240e-01, 1.98913455e-02, 1.24242112e-02, 2.01266490e+00,
        5.67616821e-01, 7.11515318e-01, 8.02313306e-02, 1.30066094e-02,
        9.66249304e-02, 9.45295053e-02, 3.02171806e-02, 5.33919058e-03,
        1.31806668e-03, 2.22997368e+01, 6.35524126e+00, 1.65239600e+02,
        8.19383712e+01, 2.19496904e+01, 7.90961186e+01, 6.15411065e-01,
        3.88141984e-02, 9.66956928e-01, 2.48999989e-02, 3.08828754e-02,
        8.85855425e-02, 3.33654254e-01, 1.75016333e-01, 4.46136918e-02,
        3.31121274e-02, 1.94237183e-01, 1.39193572e-01, 2.61722197e-02,
        6.31697785e-02, 2.82152231e-01, 2.03475111e-01, 2.01824663e-01,
        3.49230364e-02, 3.16370388e-01, 4.77140139e-02, 5.65674105e-01,
        2.96714002e-01, 4.17844331e-01, 8.23562448e-02, 2.07291774e-01,
        1.30224988e-01, 2.71120586e-02, 1.97194237e-02, 1.59319878e-01,
```

```
        4.15832846e-01, 1.81692627e-01, 7.98404566e-02, 1.35204988e-01,
        7.34105835e-03, 1.14774954e-01, 3.15132552e-01, 1.90603904e-02,
        1.91790164e-01, 1.15812216e-01])

    Survival_retrained_male = {'Qingdao': np.array([[1.        , 0.99758149, 0.99490604
, 0.99188973, 0.98885544,
         0.98484942, 0.97998834, 0.97464766, 0.96810162, 0.96082302]]),
 'Harbin': np.array([[1.        , 0.99275296, 0.98194276, 0.96929892, 0.95393163,
         0.93650829, 0.91701101, 0.89726878, 0.87276394, 0.84959334]]),
 'Haikou': np.array([[1.        , 0.99519381, 0.98985546, 0.98337945, 0.97533665,
         0.9653843 , 0.95140592, 0.9390256 , 0.92479699, 0.91083858]]),
 'Suzhou': np.array([[1.        , 0.99895283, 0.99757429, 0.99565539, 0.99294964,
         0.98927383, 0.98509091, 0.98085858, 0.97512468, 0.97013394]]),
 'Liuzhou': np.array([[1.        , 0.99622525, 0.99006084, 0.98309157, 0.97511883,
         0.96756128, 0.96064093, 0.95261631, 0.94381578, 0.93487855]]),
 'Sichuan': np.array([[1.        , 0.99815201, 0.99634497, 0.99340731, 0.99023982,
         0.98688825, 0.98285099, 0.97748382, 0.97206685, 0.96389881]]),
 'Gansu': np.array([[1.        , 0.99722033, 0.99291332, 0.98838962, 0.98324426,
         0.97616311, 0.96846008, 0.9594221 , 0.95040728, 0.93976108]]),
 'Henan': np.array([[1.        , 0.99369105, 0.9867839 , 0.97751916, 0.96647417,
         0.95386704, 0.94050813, 0.92412728, 0.9057165 , 0.88812969]]),
 'Zhejiang': np.array([[1.        , 0.99780116, 0.99430293, 0.99023676, 0.98715703,
         0.98336494, 0.9803529 , 0.97622496, 0.97243363, 0.96774917]]),
 'Hunan': np.array([[1.        , 0.99708262, 0.99223807, 0.9855671 , 0.97871828,
         0.97173772, 0.96448823, 0.95653217, 0.94690495, 0.9362808 ]])}

    S_3_male = Survival_retrained_male[region][0,3]
    S_6_male = Survival_retrained_male[region][0,6]
    S_9_male = Survival_retrained_male[region][0,9]

    M_male = male_coeffs.dot(male_means)
    x_male = risk_factor_df[['region_is_urban','age_at_study_date','sbp_mean','used_blo
od_pressure_drugs','has_diabetes',
                                'household_size','years_since_quitting_smoking','has_co
pd','hypertension_diag','tb_diag',
                                'kidney_dis_diag','children','mother_still_alive','fath
er_still_alive',
                                'mother_stroke','mother_heart_attack','father_stroke',
'father_cancer','siblings_stroke',
                                'siblings_heart_attack', 'children_diabetes','met','met
_hours','standing_height_cm',
                                'waist_cm','fat_percent','dbp_mean','smoking_category_
4',
                                'diab_under_65','sbp_noHRX','chd_diag','emph_bronc_dia
g',
                                'highest_education_0','highest_education_1','highest_ed
ucation_3','highest_education_4','highest_education_5',
                                'occupation_1','occupation_5','occupation_6',
                                'household_income_1','household_income_3','household_in
come_5',
                                'alcohol_category_1','alcohol_category_2','alcohol_cate
gory_3','alcohol_category_5',
                                'smoking_now_3',
                                'self_rated_health_1','self_rated_health_2','self_rated
_health_3',
                                'comparative_health_0','comparative_health_2','comparat
ive_health_3',
                                'diet_freq_rice_1','diet_freq_rice_2',
                                'diet_freq_wheat_0','diet_freq_wheat_3','diet_freq_whea
t_4',
                                'diet_freq_other_staple_0','diet_freq_other_staple_1',
```

```
'diet_freq_other_staple_2','diet_freq_other_staple_4',
                                'bowel_movement_freq_3',
                                'gum_bleed_freq_1','gum_bleed_freq_3']].values
    L_male = x_male.dot(male_coeffs)
    A_male = L_male-M_male
    B_male = np.exp(A_male.astype(float))

    p_male_3 = 1-(S_3_male**B_male)
    p_male_6 = 1-(S_6_male**B_male)
    p_male_9 = 1-(S_9_male**B_male)


    Cox_9yr_risk = p_male_9
    Cox_0_3yr_risk = p_male_3
    Cox_3_6yr_risk = p_male_6-p_male_3
    Cox_6_9yr_risk = p_male_9-p_male_6

else:
    #Vector format: [Age/10,smoking,CVD,65+,Diab_65-,Diab_65+,BP_drugs,sbp_noHRX,sbp_HR
X]
    female_coeffs = np.array([6.96665336e-11,  5.77980848e-01,  3.20832109e-02,  3.5539
1691e-01,
        1.79039121e-01,  4.98867265e-03,  1.83428073e-02,  1.53146246e-01,
        3.56762905e-01,  2.75490627e-03, -5.93453882e-02,  1.03446597e-01,
        6.95842226e-02,  2.70797459e-02,  2.56471893e-02,  9.14600404e-02,
        1.87796724e-01,  1.19136506e-01, -3.23469073e-03,  4.93321543e-03,
        9.49347003e-02,  9.09016705e-02,  1.23867367e-01,  1.31073856e-01,
        8.16355800e-02, -3.61649286e-03, -3.54298581e-02,  5.52890491e-02,
        1.15960518e-01, -3.93035013e-03, -8.75669035e-03,  5.79098424e-03,
        1.48342840e-02, -5.49272540e-02, -1.01230064e-01, -5.19395381e-02,
        2.52351161e-01,  4.50539320e-02,  1.18869309e-01,  1.43957997e-02,
        2.34338151e-02,  3.20059381e-02,  3.86267756e-02,  1.43017553e-02,
       -1.34810796e-01,  1.27499346e-01,  3.38979266e-02,  3.31247108e-02,
        4.44117948e-02,  2.90054817e-02,  2.03138199e-02,  2.19507659e-02,
        3.86610067e-02,  6.49412569e-02, -7.21148141e-02,  7.53941322e-02,
       -9.48817902e-02,  1.75655518e-01,  5.11219274e-02, -2.28438539e-02,
       -3.99760745e-02, -5.76750418e-03, -9.43172031e-03,  1.69251377e-02,
       -1.23996221e-01, -4.94656737e-02, -1.41487845e-02,  6.44524024e-03,
       -4.70087377e-02,  3.69585135e-02])

    female_means = np.array([4.44027175e-01, 5.13217389e+00, 1.29663272e+01, 1.14239102
e-01,
        5.96179157e-02, 3.79464152e+00, 6.13754404e-02, 1.12134801e-01,
        2.31315464e-03, 1.11638281e-02, 8.40538134e-03, 7.51243271e-02,
        1.59122972e-02, 5.57324464e-02, 2.47629706e-02, 1.34336357e-02,
        7.03797987e-03, 5.25287075e-03, 2.12710135e+00, 3.53610757e+00,
        5.33984064e-01, 6.86364604e-01, 8.20931766e-02, 9.30567025e-02,
        3.13788464e-02, 3.55926787e-02, 5.87884838e-03, 6.34442754e-04,
        1.50138316e-03, 2.05448387e+01, 1.54157595e+02, 7.89887503e+01,
        7.67066333e+01, 7.96543706e+00, 9.49800210e-01, 8.46055027e-03,
        4.32760890e-02, 6.14425494e-01, 3.04965992e-02, 2.52346650e-01,
        3.14222551e-01, 1.34840759e-01, 2.91055539e-02, 1.53251421e-02,
        1.07839506e-01, 1.74566333e-01, 1.55852242e-02, 1.95932473e-01,
        2.95173506e-01, 2.42881237e-01, 1.64982701e-01, 6.35400329e-01,
        1.40286721e-02, 2.05740722e-02, 2.75375740e-01, 1.09782240e-01,
        1.67768732e-01, 1.72639361e-01, 3.32983930e-02, 1.53188370e-01,
        3.83696003e-01, 6.61042062e-02, 2.09815342e-01, 1.13707116e-01,
        1.25757588e-01, 9.72155450e-03, 1.35049613e-01, 4.58816390e-01,
        2.33857176e-01, 7.85329792e-02])

    Survival_retrained_female = {'Qingdao': np.array([[1.        , 0.99821423, 0.996249
```

```
98, 0.99383367, 0.99144152,
         0.98850935, 0.98587218, 0.98210047, 0.97837608, 0.97385036]]),
 'Harbin': np.array([[1.        , 0.99609271, 0.98952205, 0.98094408, 0.96977076,
         0.95650486, 0.9388584 , 0.92038984, 0.89885277, 0.87822399]]),
 'Haikou': np.array([[1.        , 0.99624401, 0.99258992, 0.9866245 , 0.97799106,
         0.96676984, 0.952821  , 0.94115972, 0.92796091, 0.91520572]]),
 'Suzhou': np.array([[1.        , 0.99945132, 0.99863576, 0.99714686, 0.99553092,
         0.99324278, 0.99055756, 0.98737954, 0.98385757, 0.979502  ]]),
 'Liuzhou': np.array([[1.        , 0.99734407, 0.99410679, 0.99012963, 0.98594506,
         0.98171725, 0.97626517, 0.97049495, 0.96513527, 0.95968084]]),
 'Sichuan': np.array([[1.        , 0.99898662, 0.99769832, 0.99566427, 0.9931571 ,
         0.99020767, 0.98699539, 0.98267141, 0.97680619, 0.96910609]]),
 'Gansu': np.array([[1.        , 0.99791306, 0.99455151, 0.99005079, 0.98528677,
         0.97994769, 0.97251081, 0.96406624, 0.95339551, 0.94138531]]),
 'Henan': np.array([[1.        , 0.99534952, 0.98989205, 0.98248286, 0.97493004,
         0.96465479, 0.95166268, 0.93902037, 0.92369559, 0.90891486]]),
 'Zhejiang': np.array([[1.        , 0.99833995, 0.99609264, 0.99319939, 0.99041822,
         0.98782366, 0.98518912, 0.98201589, 0.97926997, 0.97581956]]),
 'Hunan': np.array([[1.        , 0.99706195, 0.99320991, 0.98800528, 0.98181765,
         0.97580811, 0.96908103, 0.96297801, 0.95640597, 0.94868877]])}

    S_3_female = Survival_retrained_female[region][0,3]
    S_6_female = Survival_retrained_female[region][0,6]
    S_9_female = Survival_retrained_female[region][0,9]

    #Female Model
    M_female = female_coeffs.dot(female_means)
    x_female = risk_factor_df[['region_is_urban','age_at_study_date','sbp_mean','used_b
lood_pressure_drugs','has_diabetes',
                                'household_size','has_copd','hypertension_diag','rheum_
heart_dis_diag','tb_diag',
                                'cirrhosis_hep_diag','gall_diag','kidney_dis_diag','fra
cture_diag','rheum_arthritis_diag',
                                'neurasthenia_diag','head_injury_diag','cancer_diag',
                                'children','siblings','mother_still_alive','father_stil
l_alive',
                                'mother_stroke','father_stroke','siblings_stroke','sibl
ings_diabetes',
                                'siblings_heart_attack','children_stroke', 'children_di
abetes','met','standing_height_cm',
                                'waist_cm','dbp_mean','heart_rate_mean_10s','smoking_ca
tegory_1','smoking_category_3',
                                'diab_under_65','sbp_noHRX','chd_diag',
                                'highest_education_0','highest_education_1','highest_ed
ucation_3','highest_education_4','highest_education_5',
                                'occupation_1','occupation_5','occupation_9',
                                'household_income_2','household_income_3','household_in
come_4','household_income_5',
                                'alcohol_category_1','alcohol_category_4',
                                'smoking_now_3',
                                'self_rated_health_1','self_rated_health_3',
                                'comparative_health_0','comparative_health_2','comparat
ive_health_3',
                                'diet_freq_rice_2',
                                'diet_freq_wheat_0','diet_freq_wheat_1','diet_freq_whea
t_3','diet_freq_wheat_4',
                                'diet_freq_other_staple_0','diet_freq_other_staple_1',
'diet_freq_other_staple_2','diet_freq_other_staple_3',
                                'gum_bleed_freq_1','gum_bleed_freq_3']].values
    L_female = x_female.dot(female_coeffs)
    A_female = L_female-M_female
```

```
    B_female = np.exp(A_female.astype(float))
    p_female = 1-(S_9_female**B_female)

    p_female_3 = 1-(S_3_female**B_female)
    p_female_6 = 1-(S_6_female**B_female)
    p_female_9 = 1-(S_9_female**B_female)

    Cox_9yr_risk = p_female_9
    Cox_0_3yr_risk = p_female_3
    Cox_3_6yr_risk = p_female_6-p_female_3
    Cox_6_9yr_risk = p_female_9-p_female_6
```

Next, since all of the machine learning models use one-hot encodings for region, we further process the risk factor input vector accordingly.

In [7]:

```
#For ML Models, one-hot encode region
risk_factor_df['region_Gansu'] = 0
risk_factor_df['region_Haikou'] = 0
risk_factor_df['region_Harbin'] = 0
risk_factor_df['region_Henan'] = 0
risk_factor_df['region_Hunan'] = 0
risk_factor_df['region_Liuzhou'] = 0
risk_factor_df['region_Qingdao'] = 0
risk_factor_df['region_Sichuan'] = 0
risk_factor_df['region_Suzhou'] = 0
risk_factor_df['region_Zhejiang'] = 0

risk_factor_df[('region_'+ region)] = 1

risk_factor_df.drop(['region'],axis=1, inplace=True)
```

In the following cell, we load the trained random survival forest (RSF) model objects and calculate the corresponding risk estimates.

Note: Since the RSF models are implemented using the ranger() package in R, we use rpy2 to convert the pandas dataframe to an R dataframe for compatability.

In [8]:

```
#RSF Model Output

##########################################################
#IMPORT R FUNCTIONS AND PACKAGES

from rpy2.robjects.packages import importr
# import R's "base" package
base = importr('base')
# import R's "utils" package
utils = importr('utils')
# import R's "ranger" package
ranger = importr('ranger')

import rpy2.robjects as robjects
from rpy2.robjects import pandas2ri
from rpy2.robjects.conversion import localconverter

readRDS = robjects.r['readRDS']
predict = robjects.r['predict']
which = robjects.r['which']
###########################################################
#CONVERT PANDAS RISK FACTOR DATAFRAME TO R DATAFRAME FOR INPUT TO RSF MODEL

risk_factor_df.to_csv("risk_factor_df.csv",index=False)
pd_risk_factor_df=pd.read_csv("risk_factor_df.csv")

pd_risk_factor_df=pd_risk_factor_df.rename(columns={'region_Gansu': 'Gansu',
                                  'region_Haikou': 'Haikou',
                                  'region_Harbin': 'Harbin',
                                  'region_Henan': 'Henan',
                                  'region_Hunan': 'Hunan',
                                  'region_Liuzhou': 'Liuzhou',
                                  'region_Qingdao': 'Qingdao',
                                  'region_Sichuan': 'Sichuan',
                                  'region_Suzhou': 'Suzhou',
                                  'region_Zhejiang': 'Zhejiang'})

with localconverter(robjects.default_converter + pandas2ri.converter):
    r_risk_factor_df = robjects.conversion.py2rpy(pd_risk_factor_df)
#############################################################
#CALCULATE RISK ESTIMATES

if (sex == 'Male'):
    Male_RSF_Model = readRDS("Male_RSF_Model.rds")
    survival_predictions = predict(Male_RSF_Model, r_risk_factor_df)

    prob_stroke_3 = 1-survival_predictions.rx2('survival')[which(survival_predictions.r
x2('unique.death.times').ro==3.0)[0]]
    prob_stroke_6 = 1-survival_predictions.rx2('survival')[which(survival_predictions.r
x2('unique.death.times').ro==6.0)[0]]
    prob_stroke_9 = 1-survival_predictions.rx2('survival')[which(survival_predictions.r
x2('unique.death.times').ro==9.0)[0]]

    RSF_9yr_risk = prob_stroke_9
    RSF_0_3yr_risk = prob_stroke_3
    RSF_3_6yr_risk = prob_stroke_6-prob_stroke_3
    RSF_6_9yr_risk = prob_stroke_9-prob_stroke_6

else:
```

```
    Female_RSF_Model = readRDS("Female_RSF_Model.rds")
    survival_predictions = predict(Female_RSF_Model, r_risk_factor_df)

    prob_stroke_3 = 1-survival_predictions.rx2('survival')[which(survival_predictions.r
x2('unique.death.times').ro==3.0)[0]]
    prob_stroke_6 = 1-survival_predictions.rx2('survival')[which(survival_predictions.r
x2('unique.death.times').ro==6.0)[0]]
    prob_stroke_9 = 1-survival_predictions.rx2('survival')[which(survival_predictions.r
x2('unique.death.times').ro==9.0)[0]]

    RSF_9yr_risk = prob_stroke_9
    RSF_0_3yr_risk = prob_stroke_3
    RSF_3_6yr_risk = prob_stroke_6-prob_stroke_3
    RSF_6_9yr_risk = prob_stroke_9-prob_stroke_6
```

In the following cell, we load the trained logistic regression (LR) model objects and calculate the corresponding risk estimates.

In [9]:

```
#LR 9Yr Risk Output
if (sex == 'Male'):
    Male_LR_Model_9YrRisk = load(open('Male_LR_Model_9YrRisk.pkl', 'rb'))
    LR_9yr_risk = Male_LR_Model_9YrRisk.predict_proba(risk_factor_df)[:,1]

else:
    Female_LR_Model_9YrRisk = load(open('Female_LR_Model_9YrRisk.pkl', 'rb'))
    LR_9yr_risk = Female_LR_Model_9YrRisk.predict_proba(risk_factor_df)[:,1]

#0-3Yr Risk Output
if (sex == 'Male'):
    Male_LR_Model_0_3YrRisk = load(open('Male_LR_Model_0_3YrRisk.pkl', 'rb'))
    LR_0_3yr_risk = Male_LR_Model_0_3YrRisk.predict_proba(risk_factor_df)[:,1]

else:
    Female_LR_Model_0_3YrRisk = load(open('Female_LR_Model_0_3YrRisk.pkl', 'rb'))
    LR_0_3yr_risk = Female_LR_Model_0_3YrRisk.predict_proba(risk_factor_df)[:,1]

#3-6Yr Risk Output
if (sex == 'Male'):
    Male_LR_Model_3_6YrRisk = load(open('Male_LR_Model_3_6YrRisk.pkl', 'rb'))
    LR_3_6yr_risk = Male_LR_Model_3_6YrRisk.predict_proba(risk_factor_df)[:,1]

else:
    Female_LR_Model_3_6YrRisk = load(open('Female_LR_Model_3_6YrRisk.pkl', 'rb'))
    LR_3_6yr_risk = Female_LR_Model_3_6YrRisk.predict_proba(risk_factor_df)[:,1]

#6-9Yr Risk Output
if (sex == 'Male'):
    Male_LR_Model_6_9YrRisk = load(open('Male_LR_Model_6_9YrRisk.pkl', 'rb'))
    LR_6_9yr_risk = Male_LR_Model_6_9YrRisk.predict_proba(risk_factor_df)[:,1]

else:
    Female_LR_Model_6_9YrRisk = load(open('Female_LR_Model_6_9YrRisk.pkl', 'rb'))
    LR_6_9yr_risk = Female_LR_Model_6_9YrRisk.predict_proba(risk_factor_df)[:,1]
```

In the following cell, we load the trained support vector machine (SVM) model objects and calculate the corresponding risk estimates.

Since SVM benefits from use of scaled data values, we load a trained data scaler and scale the risk factor data prior to inputting the vector into the SVM model.

In [10]:

```
#SVM 9Yr Risk Output
scaler = load(open('data_scaler.pkl','rb'))
scaled_risk_factor_df = scaler.transform(risk_factor_df)

if (sex == 'Male'):
    Male_SVM_Model_9YrRisk = load(open('Male_SVM_Model_9YrRisk.pkl', 'rb'))
    SVM_9yr_risk = Male_SVM_Model_9YrRisk.predict_proba(scaled_risk_factor_df)[:,1]

else:
    Female_SVM_Model_9YrRisk = load(open('Female_SVM_Model_9YrRisk.pkl', 'rb'))
    SVM_9yr_risk = Female_SVM_Model_9YrRisk.predict_proba(scaled_risk_factor_df)[:,1]

#0-3Yr Risk Output
if (sex == 'Male'):
    Male_SVM_Model_0_3YrRisk = load(open('Male_SVM_Model_0_3YrRisk.pkl', 'rb'))
    SVM_0_3yr_risk = Male_SVM_Model_0_3YrRisk.predict_proba(scaled_risk_factor_df)[:,1]

else:
    Female_SVM_Model_0_3YrRisk = load(open('Female_SVM_Model_0_3YrRisk.pkl', 'rb'))
    SVM_0_3yr_risk = Female_SVM_Model_0_3YrRisk.predict_proba(scaled_risk_factor_df)[:,
1]

#3-6Yr Risk Output
if (sex == 'Male'):
    Male_SVM_Model_3_6YrRisk = load(open('Male_SVM_Model_3_6YrRisk.pkl', 'rb'))
    SVM_3_6yr_risk = Male_SVM_Model_3_6YrRisk.predict_proba(scaled_risk_factor_df)[:,1]

else:
    Female_SVM_Model_3_6YrRisk = load(open('Female_SVM_Model_3_6YrRisk.pkl', 'rb'))
    SVM_3_6yr_risk = Female_SVM_Model_3_6YrRisk.predict_proba(scaled_risk_factor_df)[:,
1]

#6-9Yr Risk Output
if (sex == 'Male'):
    Male_SVM_Model_6_9YrRisk = load(open('Male_SVM_Model_6_9YrRisk.pkl', 'rb'))
    SVM_6_9yr_risk = Male_SVM_Model_6_9YrRisk.predict_proba(scaled_risk_factor_df)[:,1]

else:
    Female_SVM_Model_6_9YrRisk = load(open('Female_SVM_Model_6_9YrRisk.pkl', 'rb'))
    SVM_6_9yr_risk = Female_SVM_Model_6_9YrRisk.predict_proba(scaled_risk_factor_df)[:,
1]
```

In the following cell, we load the trained gradient boosted tree (GBT) model objects and calculate the corresponding risk estimates.

In [11]:

```
#GBT 9Yr Risk Output

if (sex == 'Male'):
    Male_GBT_Model_9YrRisk = load(open('Male_GBT_Model_9YrRisk.pkl', 'rb'))
    GBT_9yr_risk = Male_GBT_Model_9YrRisk.predict_proba(risk_factor_df)[:,1]

else:
    Female_GBT_Model_9YrRisk = load(open('Female_GBT_Model_9YrRisk.pkl', 'rb'))
    GBT_9yr_risk = Female_GBT_Model_9YrRisk.predict_proba(risk_factor_df)[:,1]

#0-3Yr Risk Output
if (sex == 'Male'):
    Male_GBT_Model_0_3YrRisk = load(open('Male_GBT_Model_0_3YrRisk.pkl', 'rb'))
    GBT_0_3yr_risk = Male_GBT_Model_0_3YrRisk.predict_proba(risk_factor_df)[:,1]

else:
    Female_GBT_Model_0_3YrRisk = load(open('Female_GBT_Model_0_3YrRisk.pkl', 'rb'))
    GBT_0_3yr_risk = Female_GBT_Model_0_3YrRisk.predict_proba(risk_factor_df)[:,1]

#3-6Yr Risk Output
if (sex == 'Male'):
    Male_GBT_Model_3_6YrRisk = load(open('Male_GBT_Model_3_6YrRisk.pkl', 'rb'))
    GBT_3_6yr_risk = Male_GBT_Model_3_6YrRisk.predict_proba(risk_factor_df)[:,1]

else:
    Female_GBT_Model_3_6YrRisk = load(open('Female_GBT_Model_3_6YrRisk.pkl', 'rb'))
    GBT_3_6yr_risk = Female_GBT_Model_3_6YrRisk.predict_proba(risk_factor_df)[:,1]

#6-9Yr Risk Output
if (sex == 'Male'):
    Male_GBT_Model_6_9YrRisk = load(open('Male_GBT_Model_6_9YrRisk.pkl', 'rb'))
    GBT_6_9yr_risk = Male_GBT_Model_6_9YrRisk.predict_proba(risk_factor_df)[:,1]

else:
    Female_GBT_Model_6_9YrRisk = load(open('Female_GBT_Model_6_9YrRisk.pkl', 'rb'))
    GBT_6_9yr_risk = Female_GBT_Model_6_9YrRisk.predict_proba(risk_factor_df)[:,1]
```

In the following cell, we load the trained multilayer perceptron (MLP) model objects and calculate the corresponding risk estimates.

Since MLP benefits from use of scaled data values, we load a trained data scaler and scale the risk factor data prior to inputting the vector into the MLP model.

In [12]:

```
#MLP 9Yr Risk Output

scaler = load(open('data_scaler.pkl','rb'))
scaled_risk_factor_df = scaler.transform(risk_factor_df)

if (sex == 'Male'):
    Male_MLP_Model_9YrRisk = load_model('Male_MLP_Model_9YrRisk.h5')
    MLP_9yr_risk = Male_MLP_Model_9YrRisk.predict(scaled_risk_factor_df)[0]

else:
    Female_MLP_Model_9YrRisk = load_model('Female_MLP_Model_9YrRisk.h5')
    MLP_9yr_risk = Female_MLP_Model_9YrRisk.predict(scaled_risk_factor_df)[0]

#0-3Yr Risk Output
if (sex == 'Male'):
    Male_MLP_Model_0_3YrRisk = load_model('Male_MLP_Model_0_3YrRisk.h5')
    MLP_0_3yr_risk = Male_MLP_Model_0_3YrRisk.predict(scaled_risk_factor_df)[0]

else:
    Female_MLP_Model_0_3YrRisk = load_model('Female_MLP_Model_0_3YrRisk.h5')
    MLP_0_3yr_risk = Female_MLP_Model_0_3YrRisk.predict(scaled_risk_factor_df)[0]

#3-6Yr Risk Output
if (sex == 'Male'):
    Male_MLP_Model_3_6YrRisk = load_model('Male_MLP_Model_3_6YrRisk.h5')
    MLP_3_6yr_risk = Male_MLP_Model_3_6YrRisk.predict(scaled_risk_factor_df)[0]

else:
    Female_MLP_Model_3_6YrRisk = load_model('Female_MLP_Model_3_6YrRisk.h5')
    MLP_3_6yr_risk = Female_MLP_Model_3_6YrRisk.predict(scaled_risk_factor_df)[0]

#6-9Yr Risk Output
if (sex == 'Male'):
    Male_MLP_Model_6_9YrRisk = load_model('Male_MLP_Model_6_9YrRisk.h5')
    MLP_6_9yr_risk = Male_MLP_Model_6_9YrRisk.predict(scaled_risk_factor_df)[0]

else:
    Female_MLP_Model_6_9YrRisk = load_model('Female_MLP_Model_6_9YrRisk.h5')
    MLP_6_9yr_risk = Female_MLP_Model_6_9YrRisk.predict(scaled_risk_factor_df)[0]
```

```
WARNING:tensorflow:From C:\Users\mchun\AppData\Roaming\Python\Python37\sit
e-packages\tensorflow\python\ops\nn_impl.py:180: add_dispatch_support.<loc
als>.wrapper (from tensorflow.python.ops.array_ops) is deprecated and will
be removed in a future version.
Instructions for updating:
Use tf.where in 2.0, which has the same broadcast rule as np.where
WARNING:tensorflow:From c:\users\mchun\anaconda3\lib\site-packages\keras\b
ackend\tensorflow_backend.py:422: The name tf.global_variables is deprecat
ed. Please use tf.compat.v1.global_variables instead.
```

Finally, we prepare a table comparing the risk estimates from all of the models and output it below.

Note that because separate models are trained for each time interval for binary classification methods (e.g., LR, SVM, GBT, MLP), the sum of risk estimates from 0-3 years, 3-6 years, and 6-9 years may not exactly match the overall 9-year risk estimte.

In [13]:

```
# Create table of risk estimates
fig, ax =plt.subplots(1,1)

col_labels = ['FSRP', 'Recalibrated \nand \nRefitted FSRP', 'CKB Cox', 'RSF', 'LR', 'SV
M', 'GBT', 'MLP']
row_labels = ['9-year Stroke Risk', 'Stroke Risk: 0-3 years', 'Stroke Risk: 3-6 years',
'Stroke Risk: 6-9 years']
table_vals = (np.array([
              [FSRP_9yr_risk[0], Recalibrated_Refitted_FSRP_9yr_risk[0], Cox_9yr_risk[0
], RSF_9yr_risk, LR_9yr_risk[0], SVM_9yr_risk[0], GBT_9yr_risk[0], MLP_9yr_risk[0]],
              [FSRP_0_3yr_risk[0], Recalibrated_Refitted_FSRP_0_3yr_risk[0], Cox_0_3yr_
risk[0], RSF_0_3yr_risk, LR_0_3yr_risk[0], SVM_0_3yr_risk[0], GBT_0_3yr_risk[0], MLP_0_
3yr_risk[0]],
              [FSRP_3_6yr_risk[0], Recalibrated_Refitted_FSRP_3_6yr_risk[0], Cox_3_6yr_
risk[0], RSF_3_6yr_risk, LR_3_6yr_risk[0], SVM_3_6yr_risk[0], GBT_3_6yr_risk[0], MLP_3_
6yr_risk[0]],
              [FSRP_6_9yr_risk[0], Recalibrated_Refitted_FSRP_6_9yr_risk[0], Cox_6_9yr_
risk[0], RSF_6_9yr_risk, LR_6_9yr_risk[0], SVM_6_9yr_risk[0], GBT_6_9yr_risk[0], MLP_6_
9yr_risk[0]]
             ]))

risk_estimate_df=pd.DataFrame(table_vals,columns=col_labels)
risk_estimate_df.update(risk_estimate_df.applymap('{:.1%}'.format))

ax.axis('tight')
ax.axis('off')
table = ax.table(cellText=risk_estimate_df.values,colLabels=risk_estimate_df.columns,ro
wLabels=row_labels,loc="center")
table.auto_set_font_size(False)
table.set_fontsize(12)
table.scale(3,4)
```

	FSRP	Recalibrated and Refitted FSRP	CKB Cox	RSF	LR	SVM	GBT	MLP
9-year Stroke Risk	0.2%	4.5%	4.1%	2.7%	4.7%	4.0%	4.2%	3.6%
Stroke Risk: 0-3 years	0.0%	0.7%	0.6%	0.6%	0.7%	1.1%	0.4%	0.8%
Stroke Risk: 3-6 years	0.1%	1.8%	1.6%	1.2%	1.8%	1.7%	2.7%	4.3%
Stroke Risk: 6-9 years	0.1%	2.0%	1.8%	0.9%	1.9%	1.1%	2.6%	2.1%
