# Supplementary material for: Stroke risk prediction using machine learning: a prospective cohort study of 0.5 million Chinese adults
Source: J Am Med Inform Assoc. 2021 May 9;28(8):1719–27. doi: 10.1093/jamia/ocab068 (PMC8324240; doi:10.1093/jamia/ocab068)
Supplement: ocab068_Supplementary_Data [file ocab068_supplementary_data.zip › Supplement 23-3-2021.docx]

**Supplementary Material for** **“Stroke risk prediction using machine learning: a prospective cohort study of 0.5 million Chinese adults”**

**Members of the China Kadoorie Biobank Collaborative Group**

***International Steering Committee:*** Junshi Chen, Zhengming Chen (PI), Robert Clarke, Rory Collins, Yu Guo, Liming Li (PI), Jun Lv, Richard Peto, and Robin Walters.

***International Co-ordinating Centre, Oxford:*** Daniel Avery, Derrick Bennett, Ruth Boxall, Fiona Bragg, Sushila Burgess, Kahung Chan, Yumei Chang, Yiping Chen, Zhengming Chen, Robert Clarke, Huaidong Du, Zammy Fairhurst-Hunter, Wei Gan, Simon Gilbert, Alex Hacker, Parisa Hariri, Michael Holmes, Andri Iona, Becky Im, Maria Kakkoura, Christiana Kartsonaki, Rene Kerosi, Garry Lancaster, Kuang Lin, John McDonnell, Iona Millwood, Qunhua Nie, Alfred Pozaricki, Paul Ryder, Sam Sansome, Dan Schmidt, Rajani Sohoni, Rebecca Stevens, Iain Turnbull, Robin Walters, Lin Wang, Neil Wright, Ling Yang, Xiaoming Yang and Pang Yao.

***National Co-ordinating Centre, Beijing:*** Zheng Bian, Yu Guo, Xiao Han, Can Hou, Chao Liu, Jun Lv, Pei Pei, Canqing Yu and Chun Li.

***Regional Co-ordinating Centres:* Qingdao** CDC: Zengchang Pang, Ruqin Gao, Shanpeng Li, Shaojie Wang, Yongmei Liu, Ranran Du, Liang Cheng, Xiaocao Tian, Hua Zhang, Yaoming Zhai, Feng Ning, Xiaohui Sun, Feifei Li. Licang CDC: Silu Lv, Junzheng Wang, Wei Hou. **Heilongjiang** Provincial CDC: Mingyuan Zou, Shichun Yan, Xue Zhou. Nangang CDC: Bo Yu, Yanjie Li, Qinai Xu, Quan Kang, Ziyan Guo. **Hainan** Provincial CDC: Ximin Hu, Jinyan Chen, Xiaohuan Wang. Meilan CDC: Min Weng, Zhendong Guo, Shukuan Wu, Yilei Li, Huimei Li. **Jiangsu** Provincial CDC: Ming Wu, Yonglin Zhou, Jinyi Zhou, Ran Tao, Jie Yang, Jian Su. Suzhou CDC: Fang Liu, Jun Zhang, Yihe Hu, Yan Lu, Liangcai Ma, Aiyu Tang, Yujie Hua, Jianrong Jin, Jingchao Liu. **Guangxi** Provincial CDC: Zhenzhu Tang, Naying Chen, Duo Liu. Liuzhou CDC: Mingqiang Li, Jinhuai Meng, Rong Pan, Qilian Jiang, Jian Lan, Yun Liu, Liuping Wei, Liyuan Zhou, Ningyu Chen, Ping Wang, Fanwen Meng, Yulu Qin, Sisi Wang. **Sichuan** Provincial CDC: Xianping Wu, Ningmei Zhang, Xiaofang Chen, Xunfu Zhong, Jiaqiu Liu, Qiang Sun. Pengzhou CDC: Guojin Luo, Jianguo Li, Xiaofang Chen, Xunfu Zhong, Jiaqiu Liu, Qiang Sun. **Gansu** Provincial CDC: Pengfei Ge, Xiaolan Ren, Caixia Dong. Maiji CDC: Hui Zhang, Enke Mao, Zhongxiao Li, Tao Wang, Xi Zhang. **Henan** Provincial CDC: Ding Zhang, Gang Zhou, Shixian Feng, Liang Chang, Lei Fan. Huixian CDC: Yulian Gao, Tianyou He, Huarong Sun, Pan He, Chen Hu, Xukui Zhang. **Zhejiang** Provincial CDC: Min Yu, Ruying Hu, Hao Wang, Weiwei Gong, Meng Wang. Tongxiang CDC: Chunmei Wang, Xiaoyi Zhang, Kaixu Xie, Lingli Chen, Dongxia Pan, Qijun Gu. **Hunan** Provincial CDC: Yuelong Huang, Biyun Chen, Li Yin, Huilin Liu, Zhongxi Fu, Qiaohua Xu. Liuyang CDC: Xin Xu, Hao Zhang, Huajun Long, and Libo Zhang.

**Supplementary Methods S1.** Follow up and coding for stroke cases

All fatal and non-fatal stroke cases were coded using the International Classification of Diseases 10^th^ revision (ICD-10) by trained medical staff, who were blinded to other personal information, with further checking and review conducted centrally by trained medical staff. All hospital-reported cases of first stroke also underwent additional clinical adjudication, involving retrieval and review of original medical records and brain imaging reports by clinical specialists in China using a bespoke web-based system. About 92% of the reported first stroke cases had their diagnosis confirmed by brain imaging (CT or MRI). Radiological reports (but not primary brain images) of reported cases of non-fatal stroke were adjudicated by Chinese neurologists using a bespoke online system.

**Supplementary Methods S2.** Data preprocessing

Data used in this study were from CKB version 17.00 including follow-up through January 1, 2018.

For the Cox model, which was stratified by 10 geographical areas, categorical variables (excluding area), were one-hot encoded, resulting in a total of 133 risk factor indicators. For all other ML models, 10 geographical areas were also one-hot encoded, resulting in a total of 143 risk markers. Missing values in both the training set and test set were imputed using the means of the available values in the training set. CKB has very few missing values. Of the 143 risk factor indicators considered in CKB, only 16 risk factors had missing values. Out of all 503,842 individuals included in the present analyses, 521 individuals had missing values for number of siblings and siblings’ medical history (stroke, heart attack, diabetes, and cancer); 817 had missing values for mother’s medical history (stroke, heart attack, diabetes, and cancer); 1,124 had missing values for father’s medical history (stroke, heart attack, diabetes, and cancer); 2 had missing values for weight and BMI; and 228 had missing values for body fat percentage. In addition to mean imputation, 3 binary risk factors were added to represent whether or not an individual was missing medical history for their mother, father, or siblings, respectively.

**Supplementary Methods S3.** Details of Cox and machine learning (ML) model development

Cox Models:

Cox models were derived separately for men and women with stratification by 10 geographic areas. For each model, separate baseline survival functions were developed for each CKB area (10 in total). The baseline hazard function (and corresponding baseline survival function) were derived from the non-parametric Breslow estimator.^1,2^ 10-fold cross-validated LASSO regularization was used (within the training set) for selecting a subset of risk factors from all CKB variables. LASSO regularization was performed separately for each model, yielding slightly different numbers of selected risk factors (66 risk factors for men, 70 risk factors for women). The specifics of the variable selection process have been previously described.^3,4^ The final Cox models for men and women were used for predictions at all time scales (within 9 years, 0-3 years, 3-6 years, and 6-9 years from baseline).

Random Survival Forest (RSF) Models:

RSF models were derived separately for men and women using the ranger() function of the ranger package^5^ version 0.12.1.

For men, number of trees was fixed at 150, and a grid search was performed with mtry values of [5, 15, 25, 40, 50] and min.node.size values of [10, 100, 1000] within the training set. Best performance in the training set was achieved with 150 trees, mtry=40, and min.node.size = 100.

For women, number of trees was fixed at 150, and a grid search was performed with mtry values of [5, 15, 25, 40, 50] and min.node.size values of [10, 100, 1000] within the training set. Best performance in the training set was achieved with 150 trees, mtry=40, and min.node.size = 100.

The final RSF models for men and women were used for predictions at all time scales (within 9 years, 0-3 years, 3-6 years, and 6-9 years from baseline).

Logistic Regression (LR) Models:

LR models were derived separately for both men and women using the LogisticRegressionCV() function of the scikit-learn toolkit^6^ version 0.19.2.

For both men and women, L1 penalty was applied with the liblinear solver, and regularization strength (10^-4^< C <10^4^) was optimized using k-fold cross validation in the training set, (k=5 folds selected based on runtime considerations).

Regularization strength was re-tuned, and new LR models derived, for each time scale (within 9 years, 0-3 years, 3-6 years, and 6-9 years from baseline).

Support Vector Machine (SVM) Models:

SVM models were derived separately for both men and women using the LinearSVC() function of the scikit-learn toolkit^6^ version 0.19.2.

For both men and women, L2 penalty was applied and squared hinge loss was specified. The regularization parameter (0.1≤C ≤10) was tuned using k-fold cross validation in the training set (k=3 folds selected based on runtime considerations). To calibrate the SVM decision function to meaningful probabilities, isotonic regression was implemented with k-fold cross-validation in the training set (k=2 folds selected based on runtime considerations).

Regularization strength was re-tuned, isotonic regression re-implemented, and new SVM models derived, for each time scale (within 9 years, 0-3 years, 3-6 years, and 6-9 years from baseline).

Gradient Boosted Tree (GBT) Models:

GBT models were derived separately for both men and women using the GradientBoostingClassifier() function of the scikit-learn toolkit^6^ version 0.19.2.

For men, learning_rate was initially fixed at 0.2, min_samples_split fixed at 1000, min_samples_leaf fixed at 100, max_depth fixed at 8, max_features set to ‘sqrt’, and subsample fixed at 0.8. A first grid search was performed, with k-fold cross validation in the training set (k=3 folds selected based on runtime considerations), to tune n_estimators with values ranging from 20 to 100. After setting n_estimators, a second grid search was performed, with 3-fold cross validation, to tune max_depth (values ranging from 1 to 10) and min_samples_split (values ranging from 500 to 1500). A third grid search was performed, with 3-fold cross validation, to tune min_samples_leaf (values ranging from 1 to 6). A fourth grid search was performed, with 3-fold cross validation, to tune max_features (values ranging from 10 to 60). A fifth grid search was performed, with 3-fold cross validation, to tune subsample (values ranging from 0.6 to 0.9). A sixth grid search was performed, with 3-fold cross valiation, to tune learning_rate (values ranging from 0.01 to 0.2) and n_estimators (values ranging from 60 to 1200). The final tuned model for men had the following hyperparameters: learning_rate = 0.02, n_estimators = 1200, max_depth = 5, min_samples_split = 900, min_samples_leaf = 1, subsample = 0.9, and max_features = 25.

For women, an identical hyperparameter methodology was employed. The final tuned model for women had the following hyperparameters: learning_rate = 0.01, n_estimators = 1200, max_depth = 7, min_samples_split = 1100, min_samples_leaf = 1, subsample = 0.9, and max_features = 30.

The final tuned hyperparameters were kept the same for predictions at each time scale (within 9 years, 0-3 years, 3-6 years, and 6-9 years from baseline), but the GBT models for men and women were retrained for each prediction task.

Multilayer Perceptron (MLP) Models:

MLP models were derived separately for both men and women using keras^7^ version 2.3.1.

For men, an architecture was first selected. To perform a binary classification task with 143 inputs, the input layer of the MLP was set at 143 nodes and the output layer set at 1 node with a sigmoid activation function. To select the number of hidden layers and the number of nodes per hidden layer, a grid search was performed with k-fold cross-validation (k=3 folds selected based on runtime considerations) over 1, 3, 5, or 10 hidden layers and 5, 10, 25, 50, 75, or 100 nodes per hidden layer. Since each model had different capacity and would reach a different level of fitting/overfitting for the same number of epochs, we used early stopping (with a 20 epoch maximum and a patience of 5 epochs) to allow all models to train until binary cross-entropy loss in the validation set was sufficiently minimized. For these tests, we used the stochastic gradient descent (SGD) optimizer with a default learning rate of 0.01, a default batch size of 32, a default “glorot_uniform” initialization of kernel weights, and a default initialization of zeros for the bias weights.


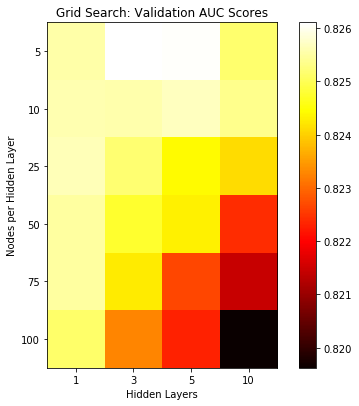


eFigure 1: Grid search for MLP architecture selection

The results of the grid search for architecture selection are shown in eFigure 1. The best performing model (as assessed by validation AUROC) had 3 hidden layers with 5 nodes each, and the worst performing model had 10 hidden layers with 100 nodes per layer. eFigure 2 shows the model accuracy and loss in the training and validation sets for both the best-performing and worst-performing models. It was observed that 20 epochs provided sufficient training for the validation loss to decrease and converge in the best-performing model, while in the worst-performing model, the model failed to improve at all due to its over-complexity.


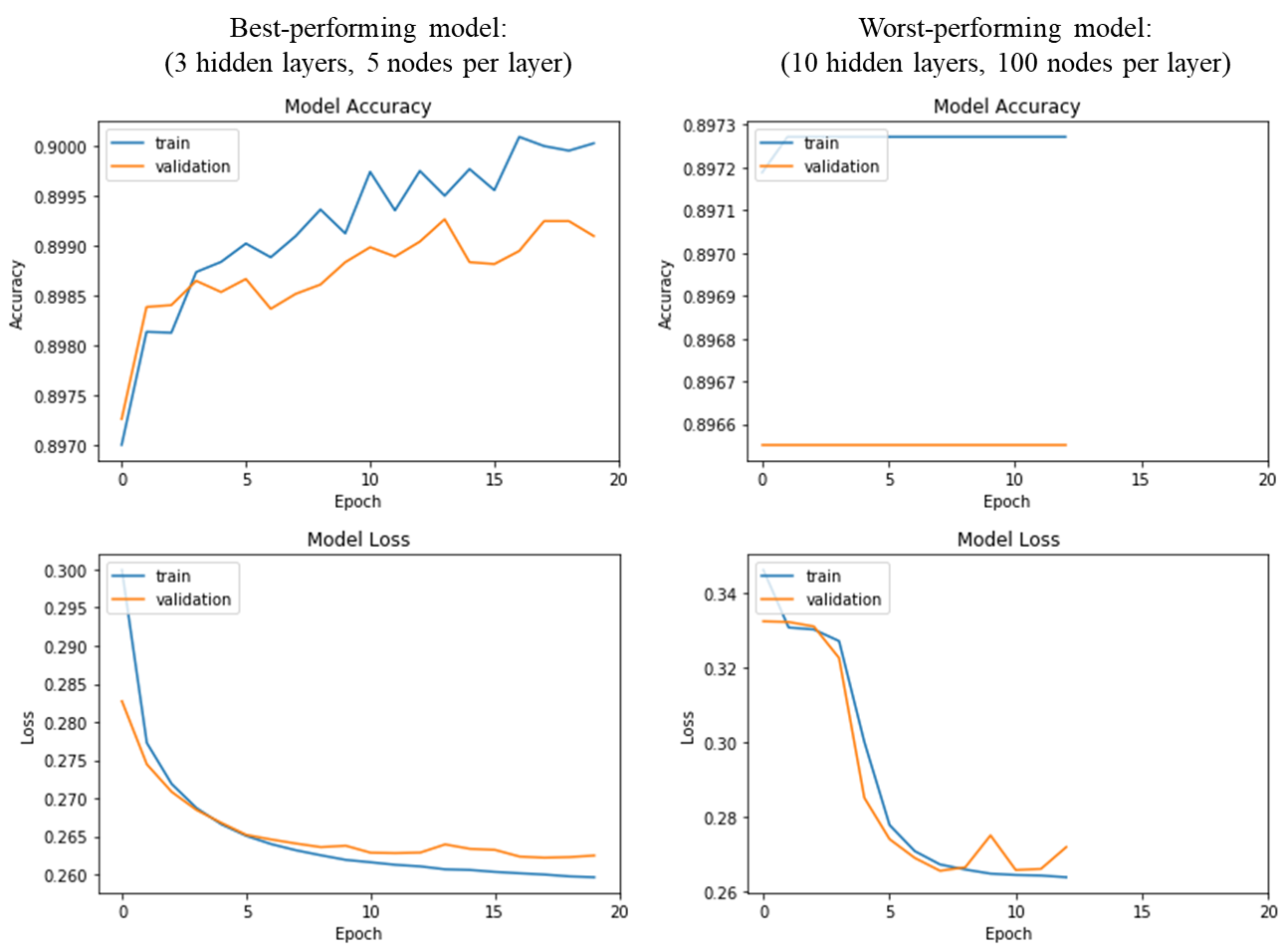


eFigure 2: Model loss and accuracy against epochs for best-performing and worst-performing model architectures

To select the best activation function for the layers of the MLP (with the exception of the output layer), a 3-fold cross-validated comparison was performed to select between ReLU, tanh, and sigmoid activation functions in the model architecture selected above. ReLU activation functions provided the best performance with a mean validation AUROC of 0.826.

Next, to find appropriate the appropriate learning rate and batch size for training the MLP, another 3-fold cross-validated grid search was performed over learning rates of 0.001, 0.01, or 0.1 and batch sizes of 32, 128, or 512. For these tests, the maximum number of epochs was increased from 20 to 50 to allow for additional training time for models with small learning rates. The best-performing model (as assessed by validation AUROC) used a learning rate of 0.1 and a batch size of 512.

To test if batch normalization would improve performance, a 3-fold cross-validated comparison was performed to select between the tuned MLP without batch normalization, with batch normalization implemented before the non-linear tanh activation function, and with batch normalization implemented after the non-linearity. A default momentum of 0.99 was used. However, batch normalization did not have any effect on the validation AUROC.

Up to this point in the hyperparameter tuning process, early stopping was used to prevent overfitting of the MLPs. However, overfitting could also be prevented with dropout, L1, or L2 regularization. Consequently, early stopping was turned off and compared with these alternative regularization techniques using a 3-fold cross-validated approach. Dropout was tested with dropout ratios of 0.1, 0.2, 0.3, 0.4, and 0.5, with a ratio of 0.1 yielding the best validation AUROC. L1 and L2 regularization were both tested with regularization factors of 0.0001, 0.001, 0.01, and 0.1. A regularization factor of 0.0001 yielded the highest validation AUROC for L1 regularization, and a regularization factor of 0.001 yielded the highest validation AUROC for L2 regularization. As shown in the results summary of eTable 1, the weakly L1-regularized model (with regularization factor of 0.0001) yielded the highest mean validation AUROC of 0.8264.

eTable 1: Validation AUROCs for various regularization techniques

|  | **Early Stop** | **Dropout** | **L1** | **L2** |
| --- | --- | --- | --- | --- |
| **Best Parameter Value** | N/A | 0.1 | 0.0001 | 0.001 |
| **Mean Validation AUROC (SD)** | 0.8260  (0.0021) | 0.8174  (0.0018) | 0.8264  (0.0013) | 0.8261  (0.0016) |

Using the above tuning process, the final MLP for men had the following hyperparameters: SGD optimizer, “glorot_uniform” initialization of kernel weights, zero initialization for bias weights, a 143 node input layer, 3 hidden layers with 5 nodes each and ReLU activation functions, a 1 node output layer with a sigmoid activation function, learning rate = 0.1, batch size = 512, and L1 regularization with a regularization factor of 0.0001.

For women, a similar hyperparameter tuning process was implemented, resulting in a final MLP with the following hyperparameters: SGD optimizer, “glorot_uniform” initialization of kernel weights, zero initialization for bias weights, a 143 node input layer, 3 hidden layers with 10 nodes each and tanh activation functions, a 1 node output layer with a sigmoid activation function, learning rate = 0.01, batch size = 32, and L2 regularization with a regularization factor of 0.0001.

The final tuned hyperparameters were kept the same for predictions at each time scale (within 9 years, 0-3 years, 3-6 years, and 6-9 years from baseline), but the MLP models for men and women were retrained for each prediction task.

Decision Tree for Selection between Cox and GBT:

Decision tree models for selection between Cox and GBT were derived separately for both men and women using the DecisionTreeClassifier() function of the scikit-learn toolkit^6^ version 0.19.2.

For men, a grid search was performed, with k-fold cross validation (k=3 folds selected based on runtime considerations), to tune max_depth (values ranging from 1 to 10), min_sample_split (values ranging from 2 to 10), and max_features (values ranging from 10 to 60). The final tuned model for men had a mean validation AUROC of 0.707 and had the following hyperparameters: max_depth = 6, max_features = 45, min_samples_split = 7. All other hyperparameters were set to default values.

For women, an identical hyperparameter methodology was employed. The final tuned model for men had a mean validation AUROC of 0.728 and had the following hyperparameters: max_depth = 6, max_features = 55, min_samples_split = 8. All other hyperparameters were set to default values.


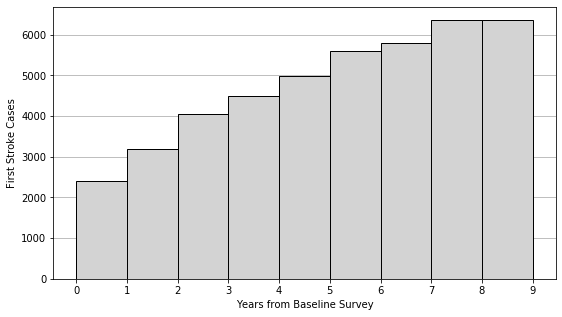


**Supplementary Figure S1.** Distribution of strokes over time from baseline survey in 503,842 adults with no prior history of stroke in the China Kadoorie Biobank.

**
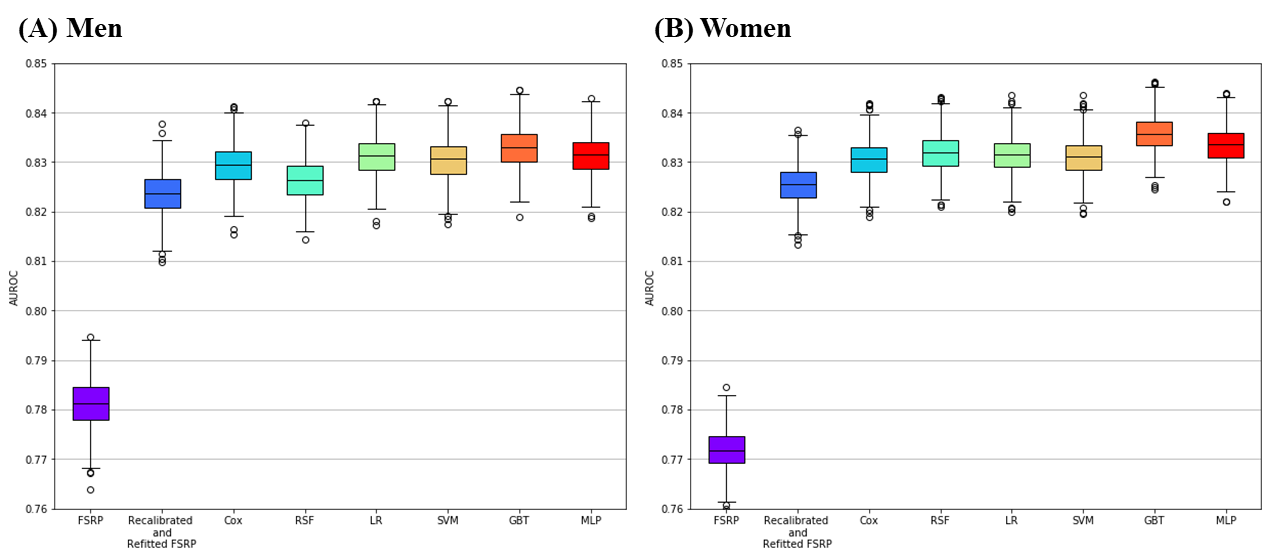
**

**Supplementary Figure S2.** AUROCs for prediction of 9 year risk of stroke (1000 bootstrapped samples from the validation set).

**
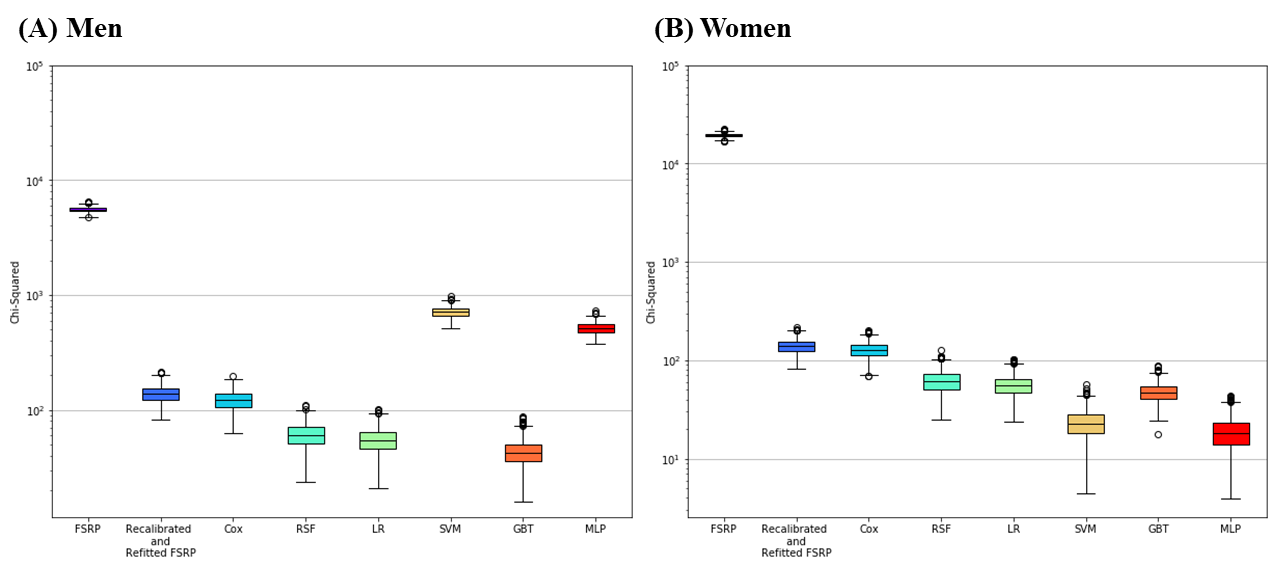
**

**Supplementary Figure S3.** Hosmer-Lemeshow/Nam-D’Agostino test statistics for prediction of 9 year risk of stroke (1000 bootstrapped samples from test set)

**
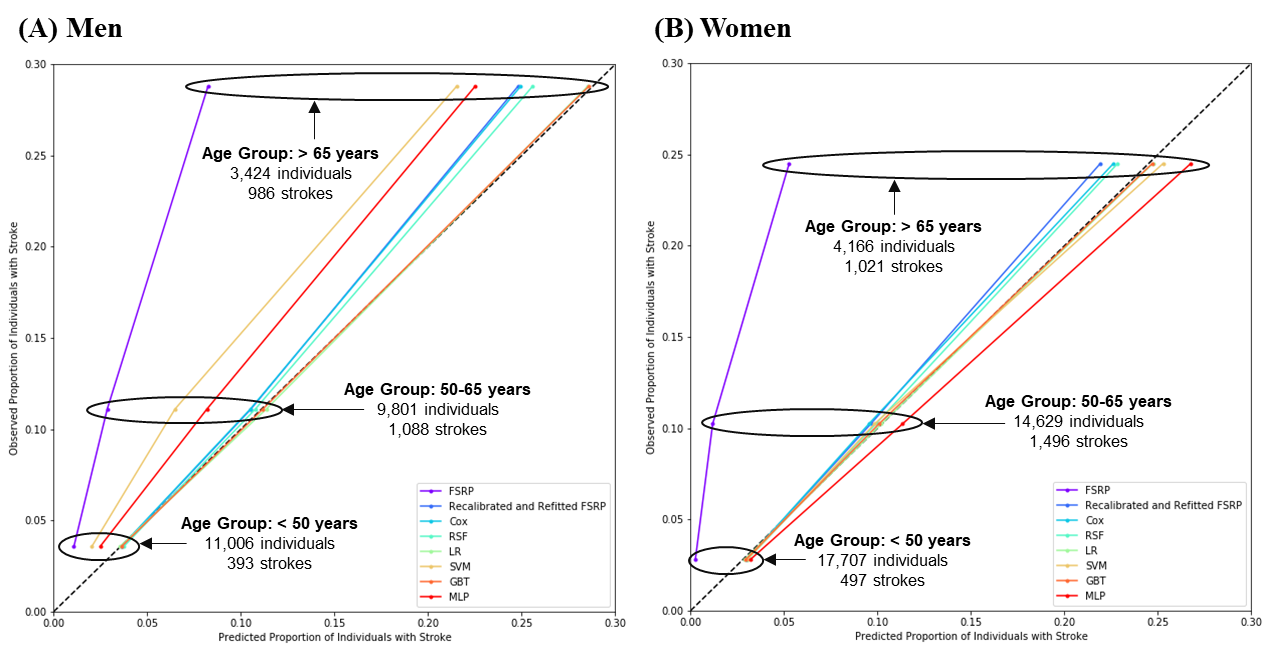
**

**Supplementary Figure S4.** Calibration plots by age group for the 2017 Framingham Stroke Risk Profile (FSRP), a recalibrated and refitted FSRP, Cox, random survival forest (RSF), logistic regression (LR), support vector machine (SVM), gradient boosted tree (GBT), and multilayer perceptron (MLP) models in (A) men and (B) women. Each point represents an age group in the CKB cohort.

**
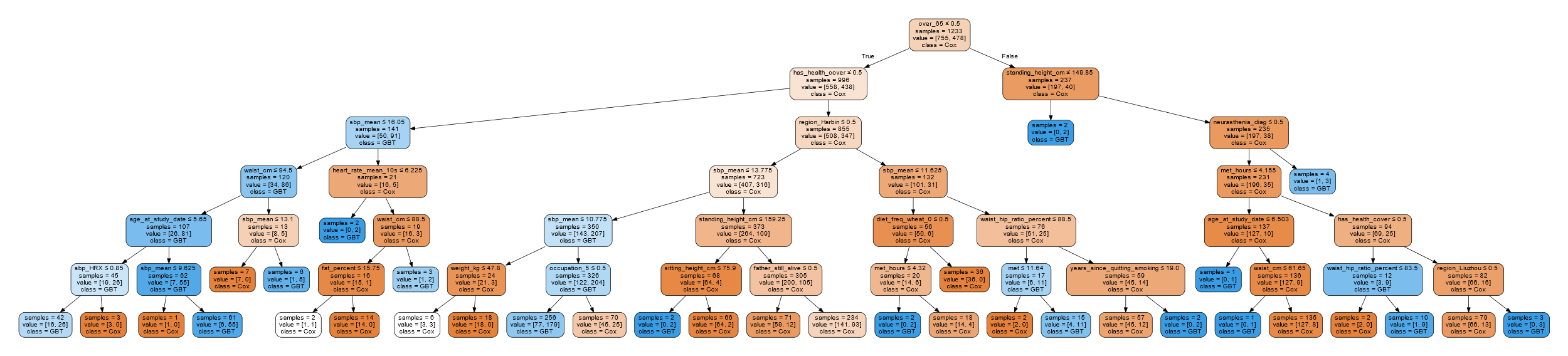
**

**Supplementary Figure S5.** Decision tree for men to select between Cox and GBT for ensemble risk screening approach.

**
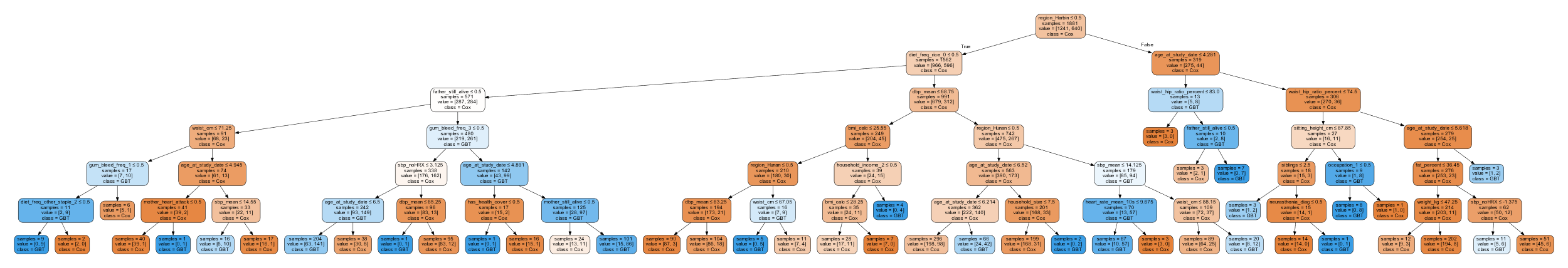
**

**Supplementary Figure S6.** Decision tree for women to select between Cox and GBT for ensemble risk screening approach.

**Supplementary Table S1.** Discrimination and Calibration performance for risk prediction of stroke from 0-3 years.

|  |  | **Men** | |  | **Women** | |
| --- | --- | --- | --- | --- | --- | --- |
| Model Type |  | Discrimination  AUROCs  [95%CI] | Calibration  χ^2^  [95%CI] |  | Discrimination  AUROCs  [95%CI] | Calibration  χ^2^  [95%CI] |
| FSRP |  | 0.800  [0.783-0.816] | 1191  [956-1443] |  | 0.803  [0.787-0.817] | 5146  [4278-6095] |
| Recalibrated and Refitted FSRP |  | 0.832  [0.818-0.846] | 51  [29-80] |  | 0.834  [0.820-0.847] | 70  [42-103] |
| Cox |  | 0.840  [0.826-0.854] | 53  [33-78] |  | 0.840  [0.826-0.852] | 62  [37-94] |
| RSF |  | 0.835  [0.821-0.848] | 26  [14-40] |  | 0.846  [0.833-0.859] | 38  [22-57] |
| LR |  | 0.867  [0.854-0.879] | 20  [9-33] |  | 0.864  [0.850-0.876] | 29  [14-52] |
| SVM |  | 0.864  [0.850-0.877] | 309  [217-433] |  | 0.862  [0.848-0.875] | 20  [9-36] |
| GBT |  | 0.864  [0.850-0.877] | 36  [20-59] |  | 0.867  [0.854-0.879] | 36  [19-60] |
| MLP |  | 0.864  [0.851-0.877] | 38  [17-69] |  | 0.865  [0.852-0.877] | 29  [13-54] |

**Supplementary Table S2.** Discrimination and Calibration performance for risk prediction of stroke from 3-6 years.

|  |  | **Men** | |  | **Women** | |
| --- | --- | --- | --- | --- | --- | --- |
| Model Type |  | Discrimination  AUROCs  [95%CI] | Calibration  χ^2^  [95%CI] |  | Discrimination  AUROCs  [95%CI] | Calibration  χ^2^  [95%CI] |
| FSRP |  | 0.768  [0.752-0.783] | 3039  [2593-3608] |  | 0.764  [0.751-0.777] | 7095  [6135-8147] |
| Recalibrated and Refitted FSRP |  | 0.810  [0.797-0.824] | 105  [65-152] |  | 0.817  [0.805-0.829] | 70  [42-103] |
| Cox |  | 0.815  [0.802-0.829] | 75  [42-118] |  | 0.822  [0.809-0.833] | 44  [23-70] |
| RSF |  | 0.814  [0.800-0.828] | 44  [23-70] |  | 0.823  [0.811-0.836] | 39  [21-61] |
| LR |  | 0.837  [0.824-0.850] | 20  [8-37] |  | 0.840  [0.829-0.852] | 23  [12-38] |
| SVM |  | 0.836  [0.824-0.849] | 353  [227-545] |  | 0.839  [0.828-0.850] | 22  [9-39] |
| GBT |  | 0.839  [0.826-0.851] | 30  [13-54] |  | 0.844  [0.833-0.855] | 24  [12-41] |
| MLP |  | 0.837  [0.824-0.885] | 386  [289-497] |  | 0.842  [0.831-0.853] | 27  [10-50] |

**Supplementary Table S3.** Discrimination and Calibration performance for risk prediction of stroke from 6-9 years.

|  |  | **Men** | |  | **Women** | |
| --- | --- | --- | --- | --- | --- | --- |
| Model Type |  | Discrimination  AUROCs  [95%CI] | Calibration  χ^2^  [95%CI] |  | Discrimination  AUROCs  [95%CI] | Calibration  χ^2^  [95%CI] |
| FSRP |  | 0.735  [0.720-0.748] | 2538  [2141-2967] |  | 0.729  [0.717-0.741] | 9655  [8505-10860] |
| Recalibrated and Refitted FSRP |  | 0.778  [0.766-0.790] | 122  [76-177] |  | 0.790  [0.780-0.801] | 128  [82-182] |
| Cox |  | 0.781  [0.769-0.793] | 146  [93-208] |  | 0.794  [0.782-0.804] | 134  [85-189] |
| RSF |  | 0.775  [0.762-0.787] | 64  [35-102] |  | 0.794  [0.784-0.805] | 65  [36-100] |
| LR |  | 0.805  [0.792-0.817] | 44  [23-69] |  | 0.810  [0.800-0.821] | 63  [37-94] |
| SVM |  | 0.805  [0.792-0.817] | 227  [157-302] |  | 0.809  [0.800-0.820] | 23  [10-41] |
| GBT |  | 0.804  [0.791-0.817] | 39  [19-64] |  | 0.815  [0.804-0.825] | 46  [25-71] |
| MLP |  | 0.804  [0.792-0.816] | 152  [97-214] |  | 0.811  [0.801-0.821] | 36  [20-56] |

**Supplementary Table S4.** Top 10 most important GBT features for risk prediction of stroke in men and women from 0-3 years, 3-6 years, and 6-9 years.

|  |  | **Men** | | |  | **Women** | | |
| --- | --- | --- | --- | --- | --- | --- | --- | --- |
| Feature Ranking |  | 0-3  Years | 3-6  Years | 6-9  Years |  | 0-3  Years | 3-6  Years | 6-9  Years |
| 1 |  | Age | Age | Age |  | Age | Age | Age |
| 2 |  | SBP | SBP | SBP |  | SBP | Harbin | Harbin |
| 3 |  | Age 65+ | Harbin | Harbin |  | Harbin | SBP | SBP |
| 4 |  | DBP | Age 65+ | Age 65+ |  | Age 65+ | Age 65+ | Age 65+ |
| 5 |  | SBP_noHRX | DBP | SBP_HRX |  | SBP_HRX | SBP_HRX | SBP_HRX |
| 6 |  | Harbin | SBP_HRX | SBP_noHRX |  | DBP | Children | Children |
| 7 |  | SBP_HRX | SBP_noHRX | Met |  | SBP_noHRX | Retired | DBP |
| 8 |  | Met | Met | DBP |  | Met | SBP_noHRX | SBP_noHRX |
| 9 |  | Phys_Activity | HTN RX | Phys_Activity |  | Phys_Activity | DBP | Met |
| 10 |  | HTN_diag | Retired | Children |  | HTN_diag | Haikou | Phys_Activity |

SBP = systolic blood pressure, DBP = diastolic blood pressure, Age 65+ is a binary variable (0 if < 65 years old, 1 otherwise), SBP_noHRX = systolic blood pressure if not on blood pressure drugs, SBP_HRX if systolic blood pressure if on blood pressure drugs, Met = daily physical activity (metabolic equivalent), Phys_Activity = daily physical activity (MET-hours), HTN_diag = hypertension diagnosis.

**Supplementary Table S5.** Top 10 most important decision tree features for selecting between Cox and GBT for ensemble risk screening approach.

|  |  | **Men** | |  | **Women** | |
| --- | --- | --- | --- | --- | --- | --- |
| Feature Ranking |  | Feature | Importance Score |  | Feature | Importance Score |
| 1 |  | SBP | 0.3035 |  | Age | 0.2053 |
| 2 |  | Age 65+ | 0.1399 |  | SBP_noHRX | 0.1164 |
| 3 |  | Has health coverage | 0.1012 |  | Harbin | 0.1045 |
| 4 |  | Retired | 0.0640 |  | SBP | 0.0814 |
| 5 |  | Standing height | 0.0590 |  | Eats rice daily | 0.0802 |
| 6 |  | Harbin | 0.0454 |  | Hunan | 0.0631 |
| 7 |  | Waist to hip ratio | 0.0355 |  | DBP | 0.0510 |
| 8 |  | Waist size | 0.0339 |  | Father alive | 0.0492 |
| 9 |  | Age | 0.0335 |  | Waist size | 0.0359 |
| 10 |  | Phys_Activity | 0.0315 |  | Never/rarely brushes teeth | 0.0315 |

SBP = systolic blood pressure, DBP = diastolic blood pressure, Age 65+ is a binary variable (0 if < 65 years old, 1 otherwise), SBP_noHRX = systolic blood pressure if not on blood pressure drugs, Met_hours = daily hours spend on physical activity. Importance scores calculated as the normalized total reduction of the criterion brought by that feature (Gini importance).

**Supplementary Table S6.** Confusion matrices for Cox-only, GBT-only, and ensemble approaches for screening of high-risk individuals in the test set.

| **Men** | | |  | **Women** | | |
| --- | --- | --- | --- | --- | --- | --- |
|  | | |  |  | | |
| **Cox-Only Approach** |  |  |  | **Cox-Only Approach** |  |  |
|  | Stroke | No Stroke |  |  | Stroke | No Stroke |
| Identified High Risk | 321 | 954 |  | Identified High Risk | 359 | 1161 |
| Identified Low Risk | 102 | 2904 |  | Identified Low Risk | 166 | 4755 |
|  |  |  |  |  |  |  |
| **GBT-Only Approach** |  |  |  | **GBT-Only Approach** |  |  |
|  | Stroke | No Stroke |  |  | Stroke | No Stroke |
| Identified High Risk | 338 | 992 |  | Identified High Risk | 388 | 1317 |
| Identified Low Risk | 85 | 2866 |  | Identified Low Risk | 137 | 4599 |
|  |  |  |  |  |  |  |
| **Ensemble Approach** |  |  |  | **Ensemble Approach** |  |  |
|  | Stroke | No Stroke |  |  | Stroke | No Stroke |
| Identified High Risk | 322 | 919 |  | Identified High Risk | 354 | 1136 |
| Identified Low Risk | 101 | 2939 |  | Identified Low Risk | 171 | 4780 |
|  |  |  |  |  |  |  |

**Supplementary Table S7.** Agreement among Cox-only, GBT-only, and ensemble approaches for screening of high-risk individuals in the test set, assessed using Cohen’s kappa (κ).

|  | **Men** | | |  | **Women** | | |
| --- | --- | --- | --- | --- | --- | --- | --- |
| Agreement between: | Cox-Only  and  GBT-Only | Cox-Only  and  Ensemble | GBT-Only  and  Ensemble |  | Cox-Only  and  GBT-Only | Cox-Only  and  Ensemble | GBT-Only  and  Ensemble |
|  |  |  |  |  |  |  |  |
| Cohen’s Kappa (κ) | 0.88 | 0.96 | 0.92 |  | 0.85 | 0.95 | 0.89 |

**References**

1. Breslow NE. Discussion of the paper by D.R. Cox. *J R Statist Soc B* 1972;34:215-16.
2. Lin DY. On the Breslow estimator. *Lifetime Data Anal* 2007;13:471-80.
3. Friedman J, Hastie T, Tibshirani R. Glmnet: lasso and elastic-net regularized generalized linear models. R package version 3.0-2. 2019. http://CRAN.R-project.org/package=glmnet. Accessed April 8, 2020.
4. Hastie T, Qian J. Glmnet vignette. 2014. http://www.web.stanford.edu/~hastie/Papers/Glmnet_Vignette.pdf. Accessed April 8, 2020.
5. Wright MN, Ziegler A. ranger: A Fast Implementation of Random Forests for High Dimensional Data in C++ and R. *Journal of Statistical Software 2017*;77:1-17.
6. Pedregosa F, Varoquaux G, Gramfort A, et al. Scikit-learn: machine learning in Python. *JMLR* 2011; 12(85):2825-30.
7. Chollet,
